# A USP8-targeting nanoamplifier synergizes with radiotherapy to remodel the immunosuppressive tumor microenvironment in lung cancer

**DOI:** 10.1016/j.mtbio.2026.103631

**Published:** 2026-09-04

**Authors:** Junjian Deng, Peiyi Luo, Kejun Li, Miaomiao Zhang, Ruxin Zhang, Zengxing Zhang, Songlin Wang, Yongfa Zheng

**Affiliations:** a Department of Oncology, Renmin Hospital of Wuhan University, Wuhan, Hubei, 430060, China; b Department of Oncology, Wuxue First People's Hospital, Wuxue, Hubei, 435401, China; c Department of Oncology, Zhongxiang City People's Hospital, Zhongxiang, Hubei, 431900, China

**Keywords:** USP8, Nanoparticle, Radiotherapy, Tumor microenvironment, Ferroptosis

## Abstract

Radiotherapy (RT) remains a cornerstone in lung cancer treatment, but its efficacy is often limited by radioresistance and an immunosuppressive tumor microenvironment (TME). Here, we identify the deubiquitinating enzyme USP8 as a key regulator of M2-like tumor-associated macrophages (TAMs) in the irradiated TME. To exploit this finding, we developed a biomimetic nanoparticle, USP8@CPMOF, which co-delivers a USP8 inhibitor and an anti-PD-L1 nanobody-coated membrane. This nanosystem specifically targets both irradiated tumor cells and TAMs, responding to the acidic and high-glutathione TME to trigger ferroptosis and release the USP8 inhibitor. *In vitro*, USP8@CPMOF enhanced RT-induced apoptosis and ferroptosis, promoted dendritic cell maturation, and reprogrammed M2 macrophages toward an M1 phenotype. *In vivo*, it improved tumor accumulation, synergized with RT to suppress tumor growth and metastasis, and robustly remodeled the TME by increasing cytotoxic T cell infiltration and reducing immunosuppressive cells. This work presents a promising nanomedicine strategy to overcome radioresistance by simultaneously targeting tumor cells and the immunosuppressive TME.

## Graphical abstract

**Figure:**
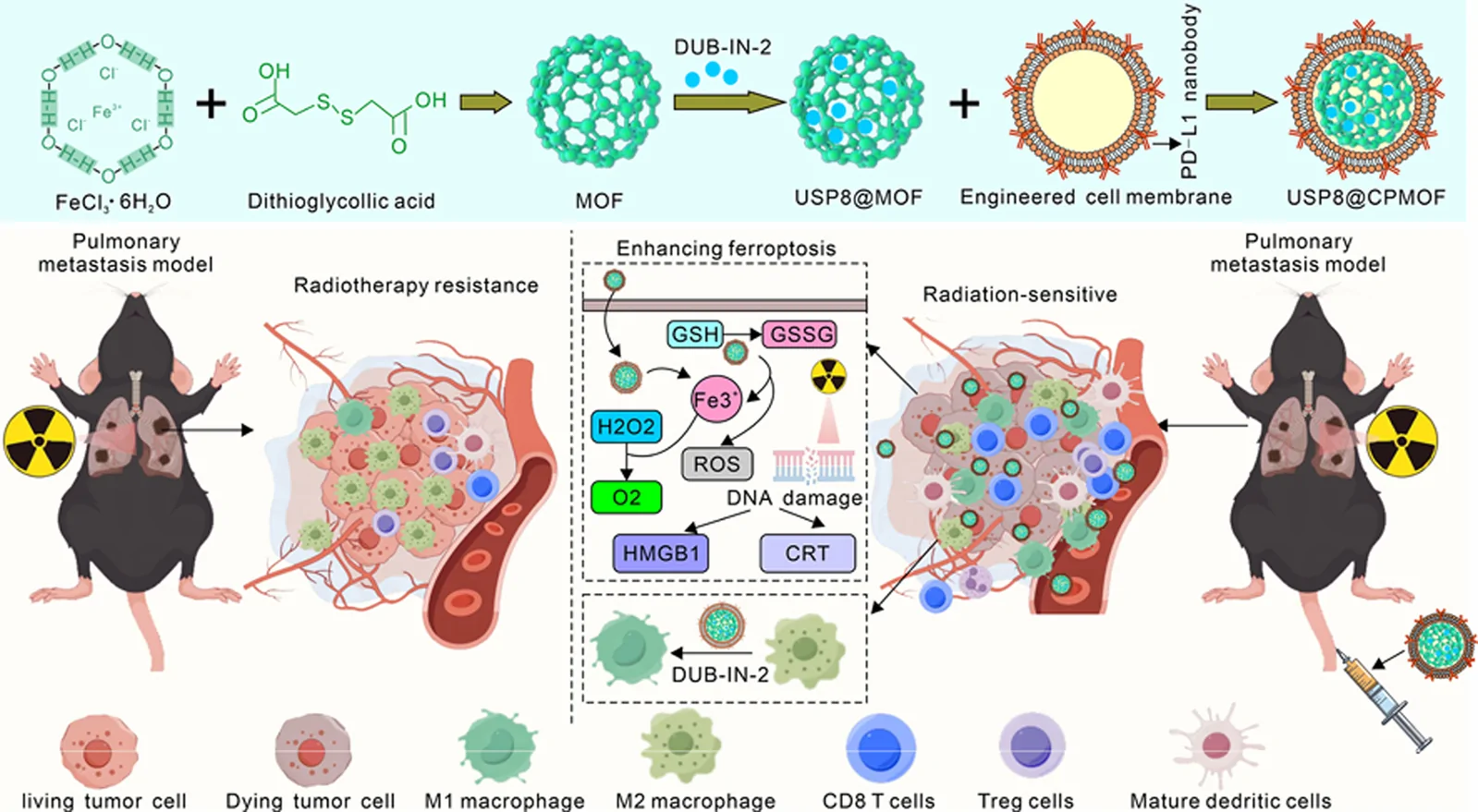

## 1 Introduction

Lung cancer remains a leading cause of cancer-related mortality globally, posing significant therapeutic challenges despite advancements in multimodal treatments, including surgery, chemotherapy, and immunotherapy [1,2]. Radiotherapy (RT) is a cornerstone in the management of both localized and advanced lung cancer, employed with curative or palliative intent in over half of all patients [3,4]. However, the clinical efficacy of RT is often constrained by intrinsic or acquired radioresistance within tumors, necessitating elevated radiation doses that risk damaging surrounding normal tissues, particularly the sensitive pulmonary parenchyma [5,6]. Consequently, there is an urgent need to develop innovative strategies that can selectively enhance the radiosensitivity of tumor cells while sparing normal tissues.

Recent evidence underscores the critical role of the tumor microenvironment (TME) in mediating radioresistance. Following irradiation, tumors frequently instigate a reparative and immunosuppressive response, characterized notably by the recruitment and polarization of tumor-associated macrophages (TAMs) towards an M2 phenotype [[7], [8], [9]]. These M2 macrophages facilitate tumor progression, promote tissue repair, and suppress antitumor immunity, thereby undermining the efficacy of radiotherapy. Therefore, disrupting the immunosuppressive TME, particularly by reprogramming M2 macrophages, represents a promising avenue to augment RT [[10], [11], [12], [13], [14]]. Concurrently, inducing novel forms of immunogenic cell death, such as ferroptosis—an iron-dependent form of regulated cell death driven by lipid peroxidation—has emerged as a potent mechanism to amplify radiation-induced cytotoxicity [[15], [16], [17]]. The acidic and redox-active TME provides an ideal niche for ferroptosis induction, where iron-based nanomaterials can catalyze the Fenton reaction, generating lethal reactive oxygen species (ROS) and synergizing with RT to cause irreversible damage to cancer cells [[18], [19], [20]].

Despite this potential, the clinical translation of radiosensitizers faces hurdles, including poor tumor-specific accumulation, limited deep-tissue penetration, and an inability to simultaneously target both cancer cells and the immunosuppressive TME. Nanotechnology offers a versatile platform to overcome these limitations. Specifically, metal-organic frameworks (MOFs) have garnered attention for their high loading capacity, tunable porosity, and potential for surface engineering [21]. When functionalized with targeting ligands and responsive to TME cues (e.g., low pH, high glutathione), such nanoparticles can achieve precise drug delivery and on-demand release [22]. Moreover, coating nanoparticles with engineered cell membranes expressing targeting moieties (e.g., anti-PD-L1 nanobodies) can enhance their homing to specific cell populations within the TME, including PD-L1^+^ tumor cells and macrophages [[23], [24], [25], [26], [27]].

In this study, we first identified the deubiquitinating enzyme USP8 as a key regulator of M2 macrophage polarization in the lung cancer TME following radiotherapy. Given its critical role in maintaining the immunosuppressive phenotype, we hypothesized that targeting USP8 could remodel the TME and enhance antitumor immunity. To translate this finding into a synergistic therapeutic strategy, we engineered a novel, multifunctional nanoparticle, USP8@CPMOF, designed to co-deliver a USP8 inhibitor (DUB-IN-2) and an anti-PD-L1 nanobody-functionalized cell membrane coating [[28], [29], [30]]. This system was engineered to be dually responsive to the acidic and high-GSH TME, enabling triggered release of the USP8 inhibitor and iron ions to concurrently induce ferroptosis in cancer cells and reprogram M2-like TAMs.

Herein, we demonstrate that USP8@CPMOF effectively targets irradiated lung tumor cells and TAMs, enhances RT-induced apoptosis and ferroptosis, promotes dendritic cell maturation and antigen presentation, and reprograms the immunosuppressive TME toward an immunostimulatory state Scheme 1. This multifunctional nanoplatform thus represents a promising approach to overcome radioresistance by simultaneously targeting cancer cells and the immunosuppressive TME, offering a potent combination strategy for the treatment of lung cancer.

**Scheme 1:**
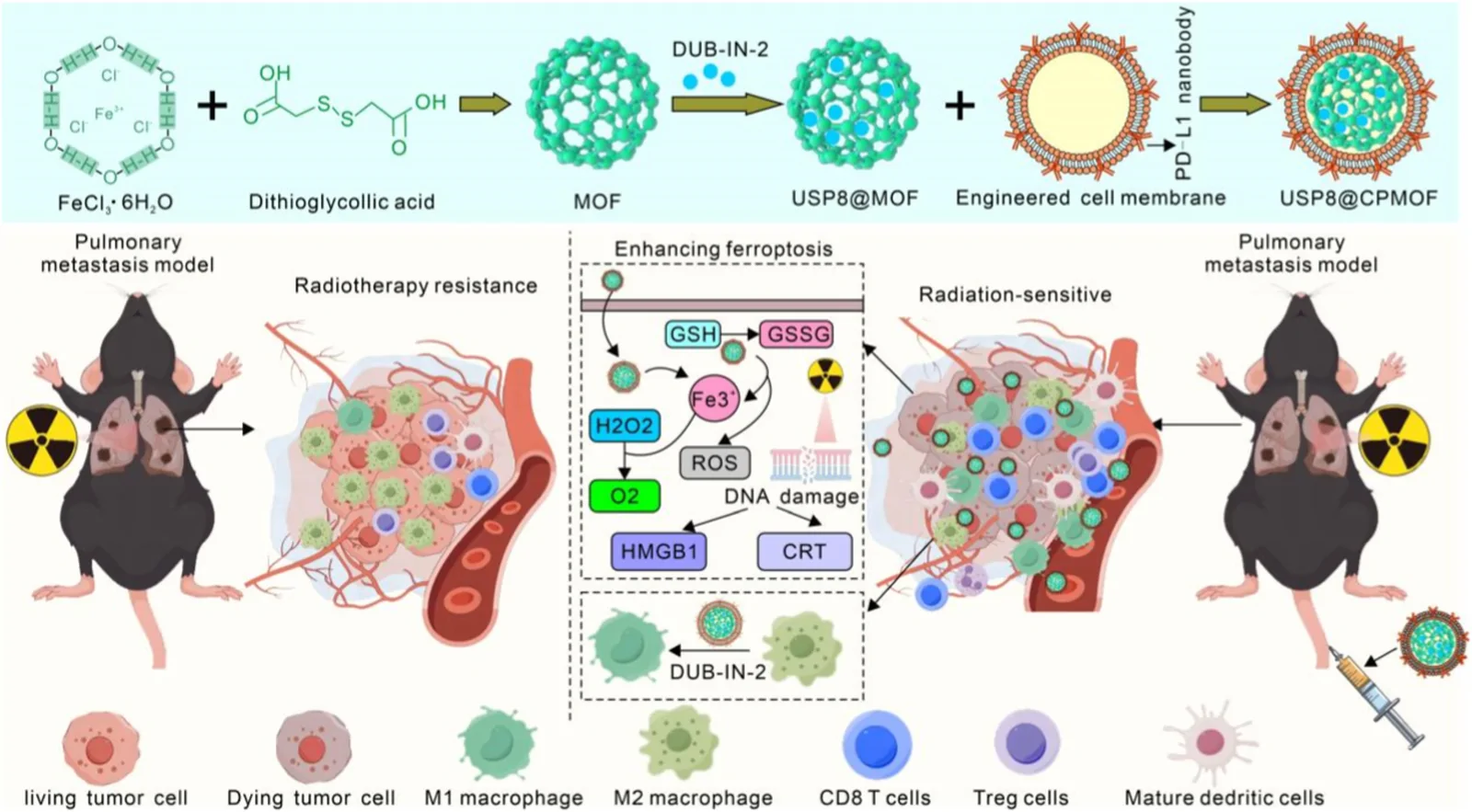
Schematic representation of the synthesis of USP8@CPMOF nanoparticles and reversal of radiation-resistant lung cancer model by synergistic ferroptosis/Macrophage reprogramming therapy/anti-PD-1 therapy.

## 2 Results

### 2.1 Identification of USP8 as a key regulator of M2 macrophage polarization in the lung cancer microenvironment

To elucidate potential deubiquitinating enzymes (DUBs) involved in modulating the radio-resistant tumor microenvironment (TME) of lung cancer, we initially profiled the expression of DUBs in tumor-associated macrophages before and after radiotherapy. This qPCR-based screening identified USP8 as a prominently differentially expressed enzyme, suggesting its potential role in the adaptive response of macrophages to radiotherapy (Fig. 1A). We next sought to characterize the expression pattern of USP8 across different macrophage polarization states. Notably, both RT-PCR and Western blot analyses revealed that USP8 expression was significantly upregulated at the mRNA and protein levels in M2-polarized bone marrow-derived macrophages (BMDMs) compared to M0 and M1 phenotypes (Fig. 1B and C). It is worth noting that the expression of USP8 protein is highly expressed only in IL-4/IL-13-induced M2-type macrophages, while its expression is very low in IL-10-induced M2-type macrophages. This indicates that IL-4/IL-13-induced M2-type macrophages are more similar to the macrophages induced in the lung cancer microenvironment after radiotherapy. This specific enrichment in BMDM derived M2 macrophages positioned USP8 as a compelling candidate regulating the immunosuppressive TME.

**Fig. 1:**
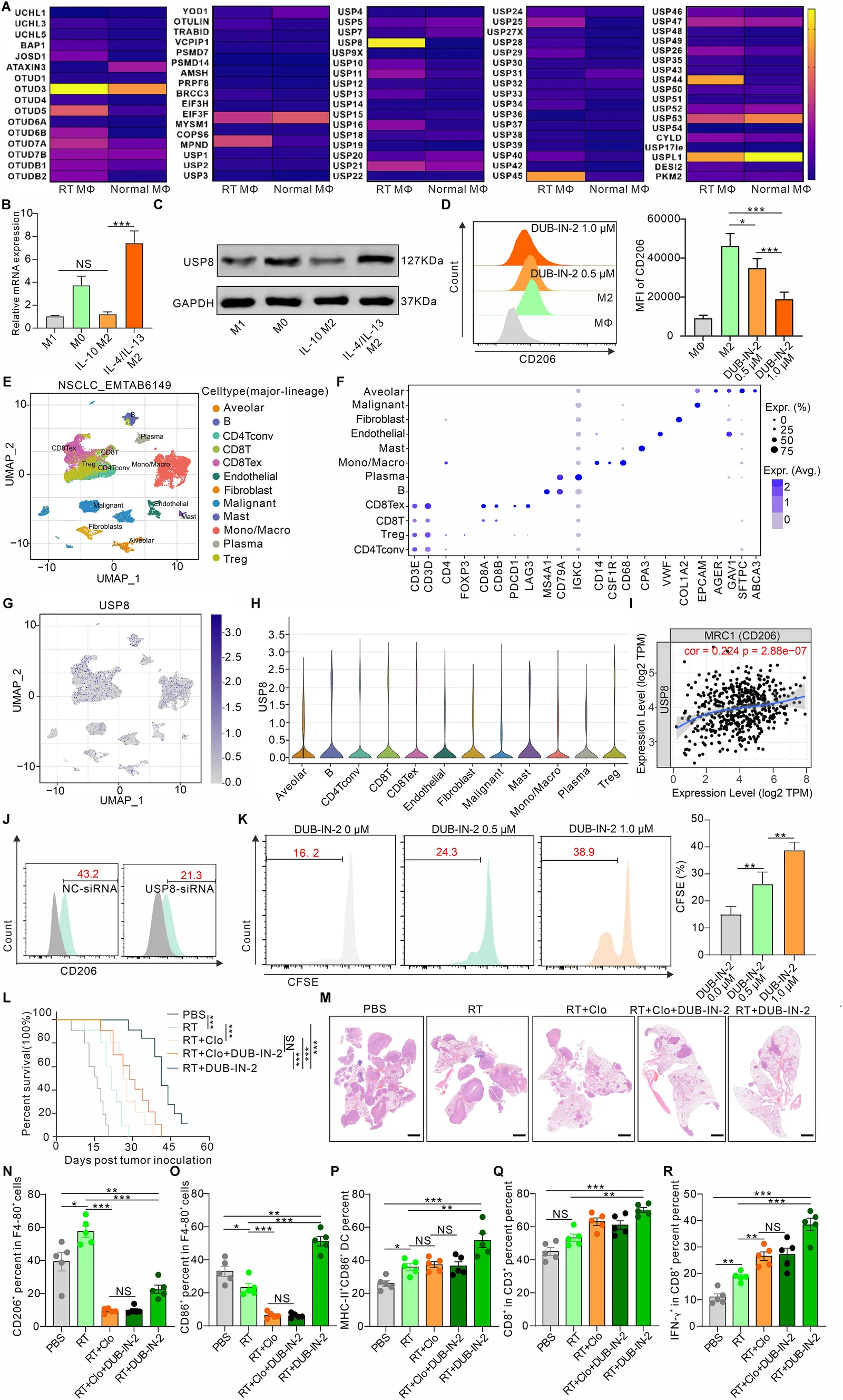
Identifying USP8 as a key target for reprogramming M2-type macrophages in the radio-resistant microenvironment of lung cancer. (A) Screening for differentially expressed deubiquitinating enzymes in macrophages from the lung cancer microenvironment before and after radiotherapy using qPCR. (B)) The mRNA expression of USP8 in M0, M1, and M2 induced from BMDMs was detected by RT-PCR. (C) Western blotting showing the expression of USP8 in M0, M1, and M2 induced from BMDMs. (D) Flow cytometry analyses of the expression of CD206 in IL-4/13-induced BMDM M2 cells, which had been treated with DUB-IN-2 (0.5 μM or 1.0 μM) for 24 h. (E) Analyzing different cell subpopulations in the lung cancer microenvironment using online single-cell databases of lung cancer (NSCLC_EMTAB6149). (F) Characterizing the gene expression profiles of different cell subpopulations in the lung cancer microenvironment. (G) Identifying the expression of USP8 in different cell subpopulations of lung cancer. (H) Statistically analyzing the average expression of USP8 in different cell subpopulations. (I) Analyzing the expression of USP8 and CD206 in different patients through the online lung cancer TCGA database. (J) The expression of CD206 in BMDMs of NC-siRNA and USP8-siRNA group was detected by flow cytometry. (K) Flow cytometry analyses of CFSE expression on the surface of CD8^+^ T cells in the presence of conditioned medium from either DMSO or DUB-IN-2 (0.5 μM or 1.0 μM) treated IL-4/13-induced BMDM M2 cells. (L) Kaplan-Meier survival plot showing survival of mice after the indicated treatments (n = 10 mice per group). (M) Representative images of H&E-stained lung tumors from different treatment groups, scale bar: 1 mm. (N-R) Percentages of CD206^+^ M2 macrophage (N), CD86^+^ M1 macrophage (O), activated DCs (P), CD8^+^ T (Q), effector CD8^+^ T cells within the TME in indicated treatment group (n = 5 mice per group). Statistical analysis was performed using one-way ANOVA with Tukey's multiple comparison test for (K and N-R). Data are represented as mean ± SEM. NS, not significant, *P < 0.05, **P < 0.01, ***P < 0.001.

To functionally validate the role of USP8 in sustaining the M2 phenotype, we treated IL-4/13-induced BMDM M2 cells with the USP8 inhibitor DUB-IN-2. Flow cytometry analysis demonstrated that pharmacological inhibition of USP8 resulted in a substantial dose-dependent reduction in the expression of the canonical M2 marker CD206 (Fig. 1D), indicating that USP8 activity is critical for maintaining M2 polarization. To contextualize our findings within the human lung cancer TME, we interrogated a public single-cell RNA sequencing database (NSCLC_EMTAB6149). Analysis of the cellular architecture confirmed distinct immune and stromal subpopulations (Fig. 1E and F). Importantly, mapping USP8 expression onto these subpopulations revealed its specific enrichment in certain cell types (Fig. 1G), which was further quantified, showing statistically significant variation across clusters (Fig. 1H). Corroborating this, an analysis of the TCGA lung cancer dataset revealed a significant positive correlation between USP8 and CD206 expression levels across patient samples (Fig. 1I), underscoring the clinical relevance of our finding. We further confirmed the necessity of USP8 for the M2 phenotype using a genetic approach. Knockdown of USP8 using specific siRNA in M2 macrophage led to a significant decrease in CD206 expression compared to the negative control group (Fig. 1J), reinforcing the conclusion that USP8 is intrinsically required for M2 macrophage polarization. To further establish the causative role of USP8 in M2 polarization, we performed gain-of-function experiments by overexpressing USP8 in BMDM M0 macrophages. Flow cytometry revealed that USP8 overexpression significantly increased the proportion of CD206^+^ M2 macrophages following IL-4/IL-13 induction (Fig. S1A, Supporting information). Western blot analysis confirmed the concurrent upregulation of USP8 and CD206 protein levels (Fig. S1B, Supporting information), providing complementary evidence that USP8 is a bona fide driver of M2 polarization. Having established USP8's role in shaping macrophage phenotype, we investigated its functional impact on T cell immunity. Conditioned medium from DUB-IN-2-treated M2 macrophages markedly enhanced the proliferation of co-cultured CD8^+^ T cells, as evidenced by reduced CFSE fluorescence (Fig. 1K). This finding suggests that inhibiting USP8 not only alters macrophage polarization but also reverses their immunosuppressive function, thereby potentiating T cell-mediated immunity.

The therapeutic potential of targeting USP8 was subsequently evaluated *in vivo*. In the lung cancer model established with lewis lung cancer (LLC) cells, treatment with a USP8-targeting agent combined with radiotherapy significantly prolonged survival (Fig. 1L) and reduced tumor burden compared with RT along or DUB-IN-2 along, as visualized by H&E-stained lung sections (Fig. 1M). Comprehensive immunophenotyping of the TME in treated mice revealed a profound reprogramming of the immune landscape: a decrease in immunosuppressive CD206^+^ M2 macrophages (Fig. 1N) was accompanied by an increase in immunostimulatory CD86^+^ M1 macrophages (Fig. 1O), activated dendritic cells (Fig. 1P), and total CD8^+^ T cells (Fig. 1Q). Most critically, we observed a significant expansion in the population of effector CD8^+^ T cells (Fig. 1R) in the combined therapy group. Collectively, these *in vivo* data demonstrated that targeting USP8 effectively remodels the TME from an radiotherapy mediated immunosuppressive to an immunostimulatory state, leading to potent anti-tumor immunity.

### 2.2 Synthesis and characterization of the multifunctional nanoparticle USP8@CPMOF

To develop a targeted and responsive drug delivery system, we synthesized a novel nanoparticle, USP8@CPMOF, designed to co-deliver the US8 inhibitor DUB-IN-2 and a cell membrane coating (highly expressed anti-PD-L1 nanobody) for enhanced tumor targeting. The schematic illustration of the synthesis process is shown in Fig. 2A. We first constructed the iron-based metal-organic framework (MOF) core, which was subsequently loaded with DUB-IN-2 to form USP8@MOF. Transmission electron microscopy (TEM) images confirmed the successful formation of USP8@MOF, revealing a uniform spherical morphology with a porous structure (Fig. 2B). Elemental mapping analysis further verified the presence and homogeneous distribution of Iron (Fe) within the nanoparticle framework, confirming the core composition (Fig. 2C). The successful loading of DUB-IN-2 into the MOF was validated by Fourier Transform Infrared (FTIR) spectroscopy. The FTIR spectrum of USP8@MOF displayed characteristic peaks corresponding to both the MOF and DUB-IN-2, indicating the effective incorporation of the drug without altering the core structure (Fig. 2D). XPS analysis was performed to confirm the stepwise construction of the nanoplatform. Full-scan spectra revealed the appearance of N 1s signals after DUB-IN-2 loading and membrane coating (Fig. S2A, Supporting information). High-resolution N1s deconvolution of USP8@MOF showed characteristic peaks at ∼398.5 eV (pyridinic N from DUB-IN-2), ∼400.0 eV (N-C), and ∼401.5 eV (protonated N). After membrane coating, USP8@CPMOF exhibited an additional peak at ∼399.0 eV corresponding to amide N from membrane proteins (Fig. S2B, Supporting information). These results confirm successful drug loading and membrane functionalization at the chemical bonding level. This was further quantified using High-Performance Liquid Chromatography (HPLC), which demonstrated a high and precise drug loading efficiency for DUB-IN-2 within the USP8@MOF construct (Fig. 2E) (The highest loading mass ratio of MOF to USP8 inhibitor was 10:1).

**Fig. 2:**
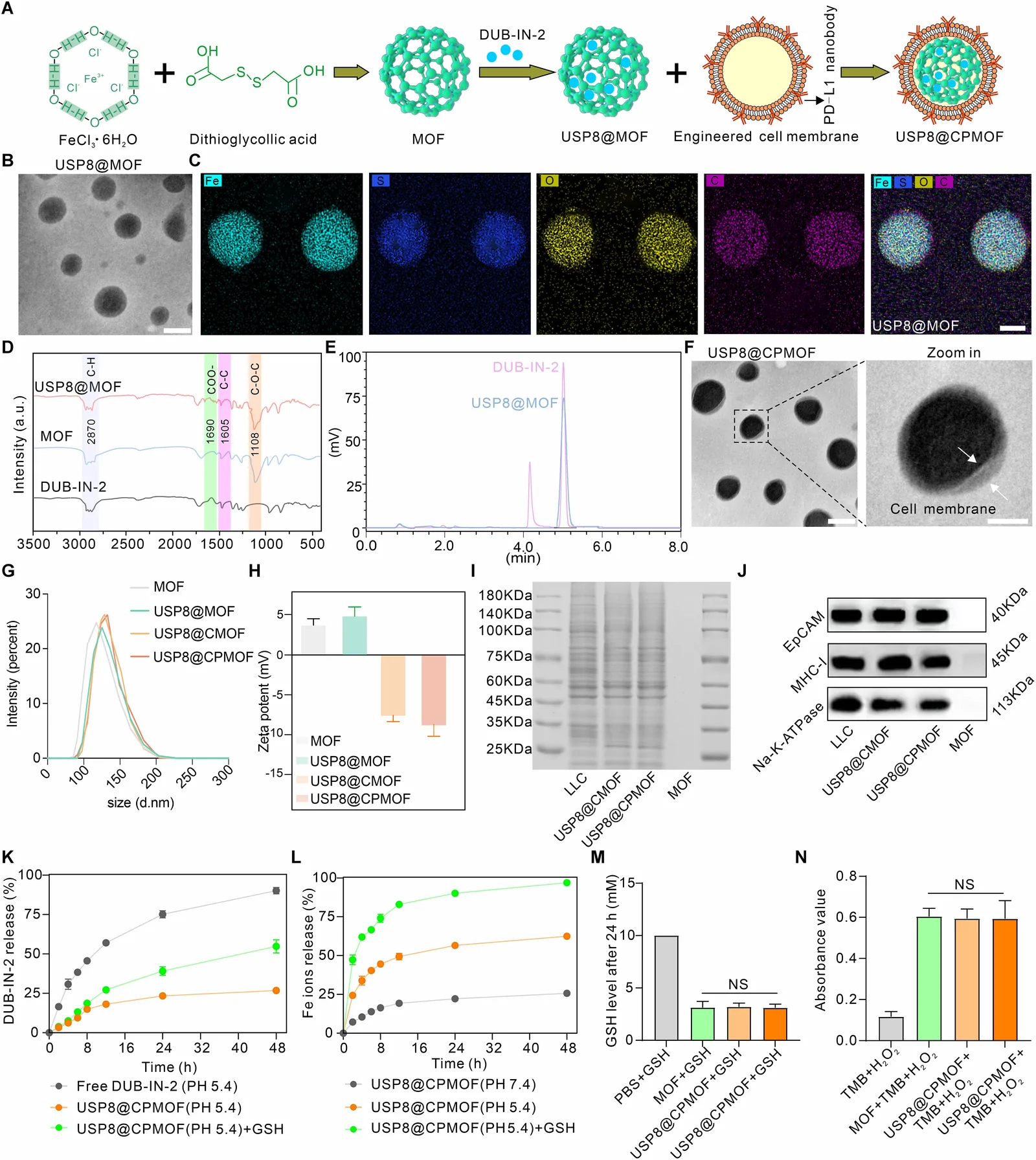
Physicochemical characterization and pH responsiveness of USP8@CPMOF. (A) Schematic diagram illustrating the synthesis process of USP8@CPMOF. (B) TEM image of USP8@MOF, the scale bar is 100 nm. (C) Mapping elemental analysis of USP8@MOF, the scale bar is 50 nm. (D) FTIR spectra of DUB-IN-2, MOF and USP8@MOF. (E) HPLC profile of DUB-IN-2 in USP8@MOF. (F) TEM image of USP8@CPMOF at different magnifications, the scale bar is 100 nm (Left) and 50 nm (Right). (G) The hydrodynamic size of MOF, USP8@MOF, USP8@CMOF and USP8@CPMOF. (H) Zeta potential of MOF, USP8@MOF, USP8@CMOF and USP8@CPMOF (n = 3). (I-J) SDS-PAGE (I) and Western blotting (J) were used to specifically detect the cell membrane components of USP8@CMOF and USP8@CPMOF. (K-L) Fe release profile (K) and DUB-IN-2 release profile (L) of USP8@CPMOF at different pH values and GSH concentration (n = 3). (M) Consumption of GSH with or without indicated formulations (n = 3). (N) Absorbance value (650 nm) changes of TMB solution with H2O2 catalyzed by indicated formulations. Data are represented as mean ± SD (NS, not significant, *P < 0.05, **P < 0.01, ***P < 0.001).

To enhance biocompatibility and active targeting, the USP8@MOF was further coated with a cell membrane derivative which highly expressed anti-PD-L1 nanobody, forming the final nanoparticle, USP8@CPMOF. To specifically verify the expression of the anti-PD-L1 nanobody on the nanoparticle surface, we constructed a GFP-tagged anti-PD-L1 nanobody fusion protein. Western blot analysis using an anti-GFP antibody revealed that both the cell membrane derivative and the final USP8@CPMOF formulation exhibited a distinct band at ∼42 kDa corresponding to the nanobody-GFP fusion protein, whereas the bare MOF showed no signal (Fig. S3, Supporting information). These results confirm the successful functionalization of the nanoparticle surface with the anti-PD-L1 nanobody. TEM images of USP8@CPMOF showed a clear core-shell structure, where the MOF core was enveloped by a thin, uniform membrane layer, confirming the successful coating process (Fig. 2F). Dynamic light scattering (DLS) analysis indicated an increase in the hydrodynamic diameter from the initial MOF (124.6 nm) to USP8@CPMOF (158.3 nm), consistent with the sequential addition of the drug and the membrane coating (Fig. 2G). The zeta potential of the nanoparticles also shifted accordingly throughout the synthesis steps, ultimately stabilizing for USP8@CPMOF, which supports the successful assembly of the final formulation (Fig. 2H). The presence of specific cell membrane proteins on USP8@CPMOF was confirmed by SDS-PAGE and Western blotting, which detected characteristic protein bands, validating the functionalization with the cell membrane components (Fig. 2I and J).

We next evaluated the responsive release profile of the therapeutic agents from USP8@CPMOF. The release of Fe ions and DUB-IN-2 was significantly accelerated in an acidic environment (pH 5.4) mimicking the tumor microenvironment (TME) compared to physiological conditions (pH 7.4). Furthermore, the addition of glutathione (GSH), which is typically overexpressed in tumor cells, drastically enhanced the release of both components, demonstrating the nanoparticle's dual responsiveness to low pH and high GSH (Fig. 2K and L). This GSH-dependent release mechanism is attributed to the degradation of the MOF framework. Accordingly, we confirmed that USP8@CPMOF could effectively consume GSH in solution, a crucial step for disrupting the intracellular redox balance and potentiating ferroptosis (Fig. 2M). Finally, we assessed the catalase-like activity of the nanoparticles, which is essential for alleviating tumor hypoxia. To evaluate the catalase-like activity of the nanoparticles, we performed UV-Vis full-wavelength scans (300-800 nm) of the TMB + H_2_O_2_ reaction system catalyzed by different formulations. The USP8@CPMOF group exhibited characteristic absorption peaks at 370 nm and 652 nm, corresponding to the oxidized TMB product, with the 652 nm absorbance being significantly higher than that of the control groups (Fig. 2N and Fig. S4A, Supporting information). Kinetic monitoring at 652 nm revealed that the catalytic reaction reached a plateau within 30 min (Fig. S4B, Supporting information), confirming the efficient H_2_O_2_-decomposing activity of USP8@CPMOF. This confirmed that the nanoparticle retained and potentially enhanced the intrinsic catalase-mimic activity of the iron-based MOF core, which is critical for generating oxygen in the TME. Collectively, these results demonstrate the successful fabrication of USP8@CPMOF, a multifunctional nanoparticle with well-characterized physicochemical properties, a responsive release profile, and inherent enzymatic activity, making it a promising platform for targeted combination therapy.

### 2.3 USP8@CPMOF potentiates radiotherapy *in vitro* through enhanced targeting and induction of apoptosis

We first investigated the cellular targeting efficiency of indicated formulations. Confocal imaging revealed that LLC cells exhibited upregulated PD-L1 expression following RT. Notably, USP8@CPMOF but not USP8@CMOF (cell membrane without expressing anti-PD-L1 nanobody) demonstrated significantly enhanced binding to LLC cells after radiation exposure, suggesting that radiotherapy augments the active targeting capability of the nanoparticle (Fig. 3A). This observation was quantitatively confirmed by flow cytometry, which showed a substantial increase in the cellular association of USP8@CPMOF in irradiated cells compared to non-irradiated controls or other formulations (Fig. 3B). To provide definitive genetic evidence for PD-L1-dependent targeting, we generated PD-L1 knockout (PD-L1 KO) LLC cells using the CRISPR-Cas9 system. Western blotting confirmed complete ablation of PD-L1 expression in PD-L1 KO cells (Fig. S5A, Supporting information). *In vitro* confocal imaging revealed that USP8@CPMOF binding to PD-L1 KO LLC cells was dramatically reduced compared to wild-type (WT) LLC cells (Fig. S5B, Supporting information).

**Fig. 3:**
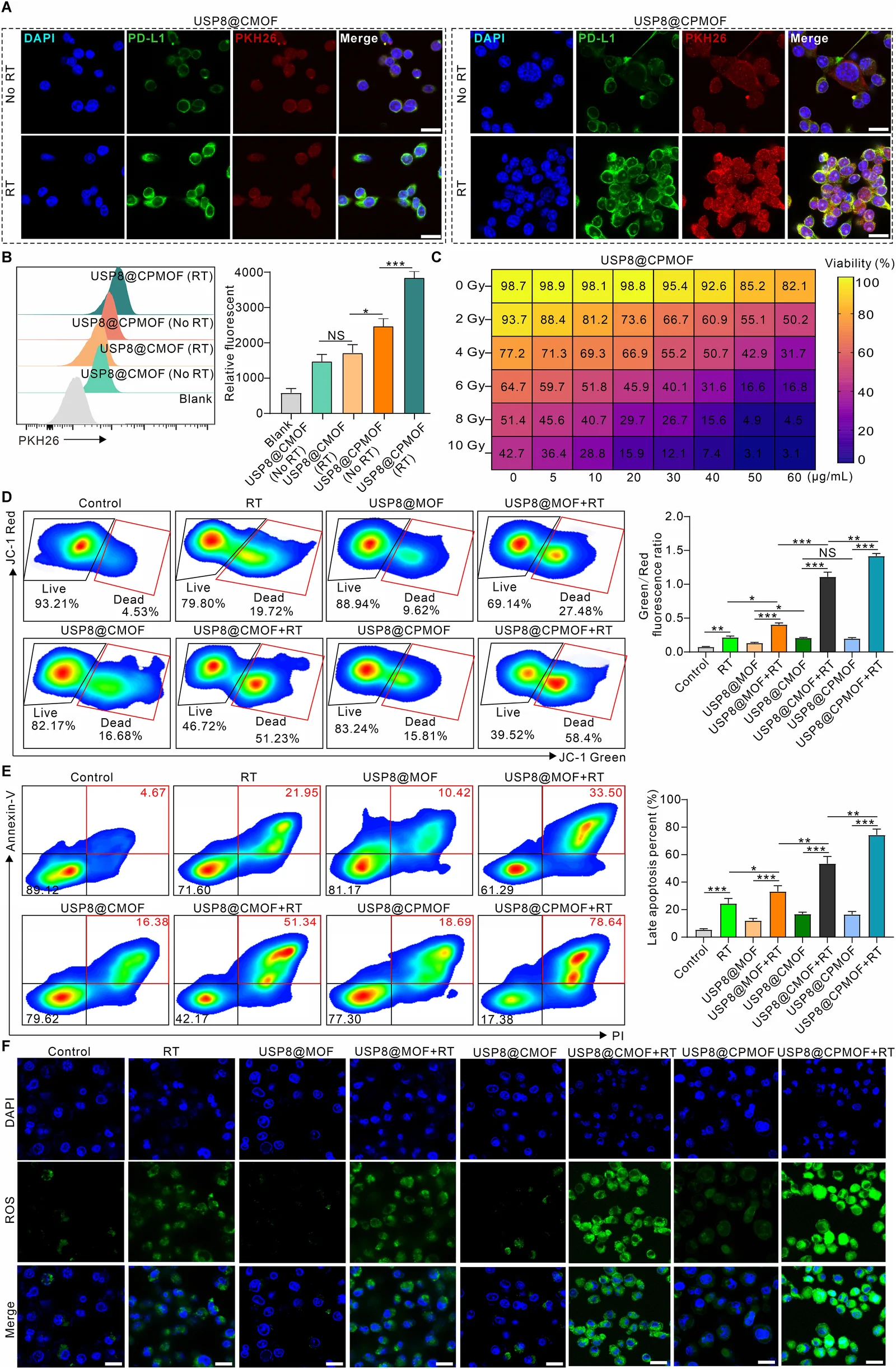
Radiosensitizing and pro-apoptotic effects of USP8@CPMOF with radiotherapy *in vitro.* (A) Representative confocal imaging of PD-L1 expression and USP8@CPMOF targeting ability of LLC cells before or after radiotherapy, the scale bar is 20 μm. (B) Flow cytometry was used to evaluate the targeting characteristics of the indicated formulations for LLC cells before and after radiotherapy (n = 3 repeats). (C) Heatmaps showing the viability of LLC tumor cells following treatment with USP8@CPMOF (0, 5, 10, 20, 30, 40, 50, and 60 μg/ml protein concentration) combined with various radiation doses (0, 2, 4, 6, 8, and 10 Gy). (D) JC-1 staining was used to assess changes in the mitochondrial membrane potential in tumor cells after different treatments. The green-to-red fluorescence ratio was quantified to assess changes in the mitochondrial membrane potential in LLC tumor cells (n = 3 repeats). (E) Annexin-V/PI staining was performed to analyze apoptosis in LLC tumor cells following different treatments (n = 3 repeats). (F) Confocal microscopy was used to observe changes in ROS levels in LLC cells after different treatments, the scale bar is 20 μm. Statistical analysis was performed using one-way ANOVA with Tukey's multiple comparison test. Data are represented as mean ± SEM (NS, not significant, *P < 0.05, **P < 0.01, ***P < 0.001). (For interpretation of the references to colour in this figure legend, the reader is referred to the Web version of this article.)

To evaluate the radiosensitizing effect of USP8@CPMOF, we treated LLC cells with varying concentrations (protein content) of the nanoparticle combined with a range of radiation doses. The resulting cell viability heatmaps demonstrated that USP8@CPMOF potently enhanced the cytotoxic effect of radiotherapy in a concentration-and dose-dependent manner (Fig. 3C). The most significant reduction in cell viability was achieved with higher nanoparticle concentrations combined with moderate to high radiation doses, indicating a strong synergistic interaction.

To rigorously exclude non-specific effects of individual components, we first evaluated the direct cytotoxicity and immunomodulatory activity of the USP8 inhibitor, the empty MOF carrier, and the cell membrane coating alone. None of these components exhibited significant antitumor activity against LLC cells or altered M2 macrophage polarization markers (Fig. S6, Supporting information). These results confirm that the MOF carrier is essential for drug delivery, the cell membrane coating enhances pharmacokinetic performance, and the anti-PD-L1 nanobody provides active targeting that translates into a significant survival advantage. Accordingly, we selected USP8@CMOF and USP8@CPMOF as the optimal nanocarrier platforms for combination with radiotherapy.

We then delved into the mechanistic basis of this radiosensitization. A key event in the intrinsic apoptosis pathway is the loss of mitochondrial membrane potential (ΔΨm). Compared with USP8@MOF or USP8@CMOF, we found that the combination of USP8@CPMOF and radiotherapy (USP8@CPMOF + RT) induced the most profound mitochondrial depolarization by using JC-1 staining, as evidenced by the highest green-to-red fluorescence ratio among all treatment groups (Fig. 3D). This suggests that the cell membrane targeting ability and PD-L1 targeting nanobody synergistically potentiates RT-induced mitochondrial damage. Consistent with the disruption of ΔΨm, apoptosis analysis by Annexin V/PI staining revealed that USP8@CPMOF + RT treatment triggered the most robust apoptotic cell death. The total apoptosis rate in the combination group was significantly higher than that in the control, RT-alone, USP8@MOF + RT, USP8@CMOF + RT or USP8@CPMOF-alone groups (Fig. 3E), confirming that the nanoparticle sensitizes tumor cells to radiation-induced apoptosis.

A primary mechanism of radiosensitization is the amplification of reactive oxygen species (ROS) generation. Confocal microscopy analysis demonstrated that intracellular ROS levels were markedly elevated in the USP8@CPMOF + RT group compared to all other treatments (Fig. 3F). This indicates that USP8@CPMOF acts as a potent radiosensitizer by exacerbating RT-induced oxidative stress, leading to irreversible mitochondrial dysfunction and, ultimately, apoptotic cell death. Collectively, these *in vitro* findings demonstrate that USP8@CPMOF effectively targets irradiated tumor cells, synergizes with radiotherapy to inhibit cell proliferation, and augments radiation-induced cytotoxicity by amplifying ROS generation and activating the mitochondrial apoptosis pathway.

### 2.4 USP8@CPMOF combined with radiotherapy induces ferroptosis and promotes antigen presenting cell activation

To delineate the role of ferroptosis in the therapeutic efficacy of USP8@CPMOF combined with radiotherapy (RT), we first assessed key hallmarks of this iron-dependent cell death pathway. To visualize ferroptosis at the cellular level, we performed C11-BODIPY 581/591 staining to detect lipid peroxidation. Confocal microscopy revealed that the USP8@CPMOF + RT group exhibited a clear shift from red (reduced) to green (oxidized) fluorescence, indicating elevated lipid peroxidation, which was significantly reversed by Ferrostatin-1 (Fer-1) pre-treatment (Fig. S7A, Supporting information). FerroOrange staining further showed a marked increase in intracellular labile Fe^2+^ in the combination group, which was not affected by Fer-1 (as Fer-1 acts downstream of iron accumulation) (Figure S7B, Supporting information). Flow cytometry analysis using the Liperfluo probe confirmed that the combination treatment induced the most significant accumulation of lipid peroxides (Fig. 4A). Concurrently, measurement of intracellular Fe^2+^ levels using Phen Green SK diacetate (PGSK) showed a substantial increase in labile iron pools specifically in the USP8@CPMOF + RT group (Fig. 4B). More importantly, pre-treatment with the ferroptosis-specific inhibitor Fer-1 significantly rescued cell viability and reduced lipid ROS levels in the USP8@CPMOF + RT group (Figure S7C-D, Supporting information), confirming that ferroptosis is a critical mechanism underlying the cytotoxic synergy. This elevation in Fe^2+^ is critical for catalyzing the Fenton reaction, thereby amplifying oxidative stress and driving lipid peroxidation. These findings confirm that the combination therapy robustly triggers ferroptosis in tumor cells. We next investigated whether this specific cell death modality could stimulate immunogenic cell death (ICD), a process that converts tumor cell death into an in situ vaccine. Confocal microscopy demonstrated that USP8@CPMOF + RT treatment potently induced the translocation of calreticulin (CRT) from the endoplasmic reticulum to the cell surface, a key "eat-me" signal for phagocytic cells (Fig. 4D). Furthermore, the same treatment prompted the pronounced release of High Mobility Group Box 1 (HMGB1) from the nucleus into the extracellular space (Fig. 4C). The robust emission of these damage-associated molecular patterns (DAMPs) indicates the induction of a highly immunogenic form of cell death.

**Fig. 4:**
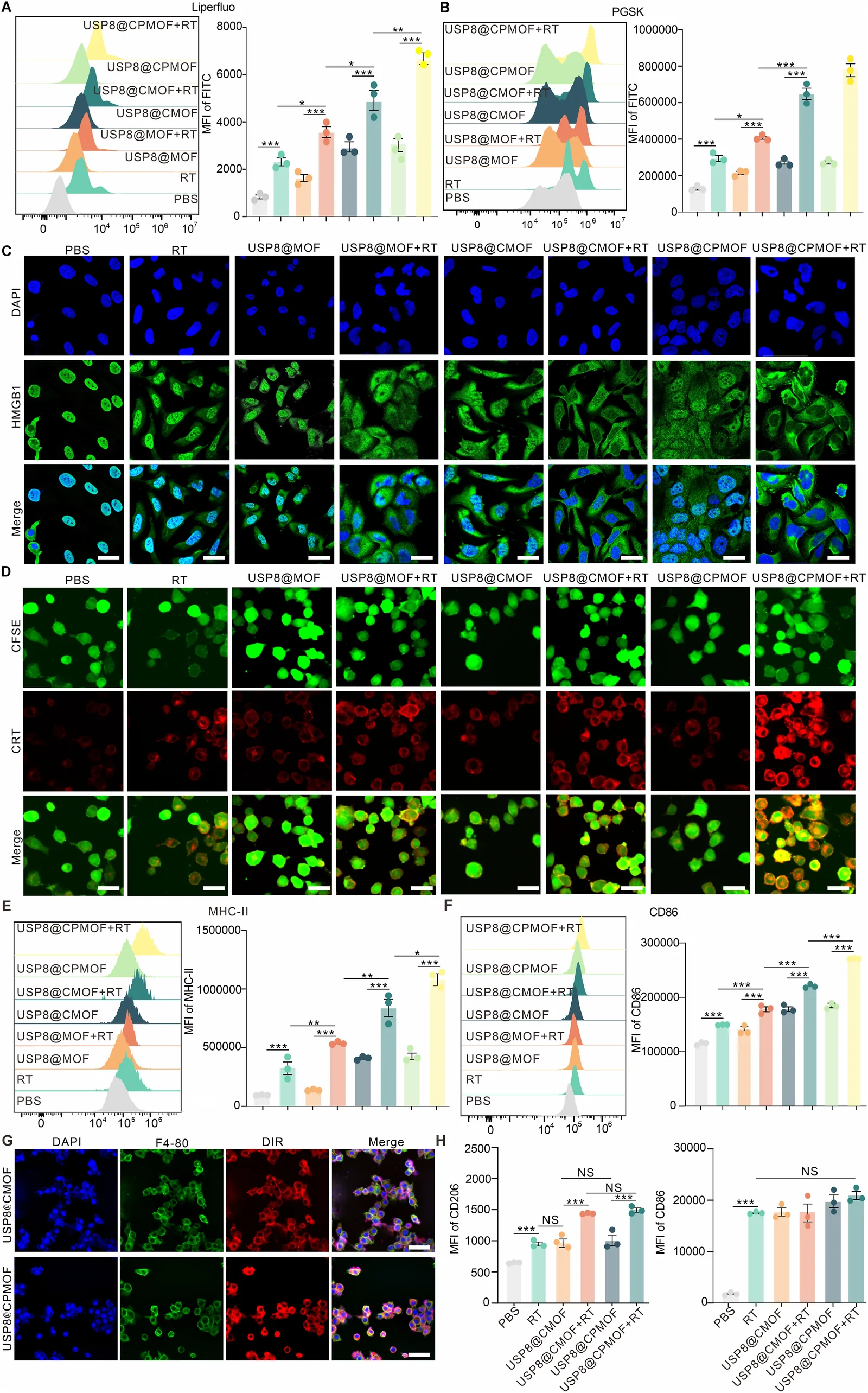
Ferroptosis induction and activation of antigen-presenting cells by indicated formulations combined with radiotherapy. (A) Flow cytometry combined with FITC-Liperfluo (5 μM) was used to measure the level of lipid peroxidation in indicated formulations (n = 3 repeats). (B) Flow cytometry combined with Phen Green SK diacetate (PGSK) (10 μM) was used to measure the level of Fe^2+^ ions in indicated formulations (n = 3 repeats). (C) Confocal microscopy image showing HMGB1 (green) release and its spatial relationship with the nucleus (blue) in LLC cells subjected to different treatments, and the scale bar is 10 μm. (D) Confocal images of CRT (Red) translocation to the cell membrane following various treatments, and the scale bar is 20 μm. (E) Flow cytometry was used to assess the effect on MHC-II expression in DCs after incubation with the supernatant of LLC cells treated with indicated formulations (n = 3 repeats). (F) Flow cytometry was used to assess the effect on CD86 expression in DCs after incubation with the supernatant of LLC cells treated with indicated formulations (n = 3 repeats). (G) Confocal imaging was used to identify the targeting ability of USP8@CMOF and USP8@CPMOF for IL-4/IL-13-induced M2-type macrophages derived from BMDM, and the scale bar is 50 μm. (H) Flow cytometry was used to evaluate the effects of indicated formulations combined with radiotherapy on the expression of CD206 and CD86 in M2-type macrophages (n = 3 repeats). Statistical analysis was performed using one-way ANOVA with Tukey's multiple comparison test. Data are represented as mean ± SEM (NS, not significant, *P < 0.05, **P < 0.01, ***P < 0.001). (For interpretation of the references to colour in this figure legend, the reader is referred to the Web version of this article.)

The critical consequence of ICD is the activation of antigen-presenting cells. To evaluate this, we incubated bone marrow-derived dendritic cells (DCs) with supernatants from indicated formulations treated LLC cells. Flow cytometry analysis revealed that supernatants from the USP8@CPMOF + RT group most effectively promoted DC maturation, as evidenced by significant upregulation of the antigen-presenting molecule MHC-II and the co-stimulatory marker CD86 (Fig. 4E and F). This indicates that the combination therapy enhances the capacity of DCs to prime adaptive anti-tumor T cell responses. Finally, we assessed the direct impact of our targeted nanoparticle on BMDM derived M2 macrophages. Confocal imaging confirmed the superior targeting ability of the cell membrane (expressing anti-PD-L1 nanobody)-coated USP8@CPMOF towards M2-type macrophages compared to the normal cell membrane formulation (Fig. 4G). When combined with radiotherapy, USP8@CPMOF significantly reprogrammed the immunosuppressive M2 phenotype, as indicated by a marked downregulation of the M2 marker CD206 and a concurrent upregulation of the immunostimulatory M1 marker CD86 (Fig. 4H). To confirm functional reprogramming, we measured M1-associated effector molecules. ELISA revealed that TNF-α and IL-1β secretion was significantly elevated in the USP8@CPMOF + RT group compared to controls (Fig. S8A–B, Supporting information). Western blot analyses further confirmed the upregulation of iNOS protein levels (Fig. S8C, Supporting information). These results provide strong evidence that USP8@CPMOF combined with RT effectively reprograms immunosuppressive M2 macrophages into pro-inflammatory M1-like cells. This demonstrates that the nanoparticle can directly remodel the tumor immune microenvironment by driving TAMs towards an anti-tumor phenotype. In summary, these findings demonstrate that USP8@CPMOF synergizes with radiotherapy not only by directly inducing ferroptosis in cancer cells but also by triggering ICD and promoting the activation and maturation of dendritic cells, thereby bridging innate and adaptive immunity. Furthermore, it directly reprograms immunosuppressive macrophages, creating a more favorable microenvironment for anti-tumor immunity.

### 2.5 USP8@CPMOF demonstrates effective tumor targeting and potent anti-tumor efficacy *in vivo*

Having validated the radiosensitizing and immunomodulatory effects of the targeted USP8@CPMOF *in vitro*, we next evaluated its biodistribution and tumor-targeting efficacy *in vivo*. To assess the targeting specificity, mice bearing LLC tumors were intravenously injected with fluorescently labeled USP8@CPMOF or control formulations, and their distribution was monitored. Representative *ex vivo* fluorescence images of major organs at 6, 12, and 24 h post-injection revealed a distinct accumulation pattern. While all formulations were detectable in the reticuloendothelial system, the USP8@CPMOF group exhibited a notably stronger and more persistent fluorescence signal specifically in the lungs (the site of LLC tumor implantation) compared to the other groups (Fig. 5A). Quantitative analysis of the radiant efficiency confirmed this observation, showing a significantly higher accumulation of USP8@CPMOF in lung tissue over the 24-h period (Fig. 5B). We further confirmed that this enhanced accumulation occurred in a context of induced target expression, as immunohistochemistry verified radiotherapy-mediated upregulation of PD-L1 in the lung tumors (Fig. 5C). To further prove this conclusion, we used LLC cells with PD-L1 knockout to construct a lung metastasis model and *ex vivo* fluorescence imaging demonstrated that USP8@CPMOF accumulation in PD-L1 KO tumors was significantly lower than in WT tumors (Fig. S9, Supporting information). To visualize cell-type-specific engagement within the tumor microenvironment, we performed immunofluorescence staining on lung sections. Consistent with the biodistribution data and the induced target expression, we observed a markedly greater co-localization of USP8@CPMOF with both EpCAM^+^ tumor cells (Fig. 5D) and F4/80^+^ macrophages (Fig. 5E) in irradiated tumors compared to non-irradiated controls. This demonstrates that the functional cell membrane coating, combined with the radiation-induced priming of the tumor microenvironment, effectively enhances the homing and retention of the nanoparticle by its intended cellular targets *in vivo*. To evaluate the *in vivo* safety of USP8@CPMOF, we monitored body weight, serum biochemistry, and major organ histology. Throughout the 21-day treatment period, no significant weight loss was observed in any group (Fig. S10, Supporting information). Serum levels of ALT, AST, BUN, and CREA remained within normal ranges and showed no significant differences between the USP8@CPMOF + RT group and the PBS control (Fig. S11A–D, Supporting information). H&E staining of heart, liver, spleen, and kidneys revealed no discernible histopathological changes (Fig. S11E, Supporting information), collectively demonstrating the favorable biosafety profile of our nanoplatform.

**Fig. 5:**
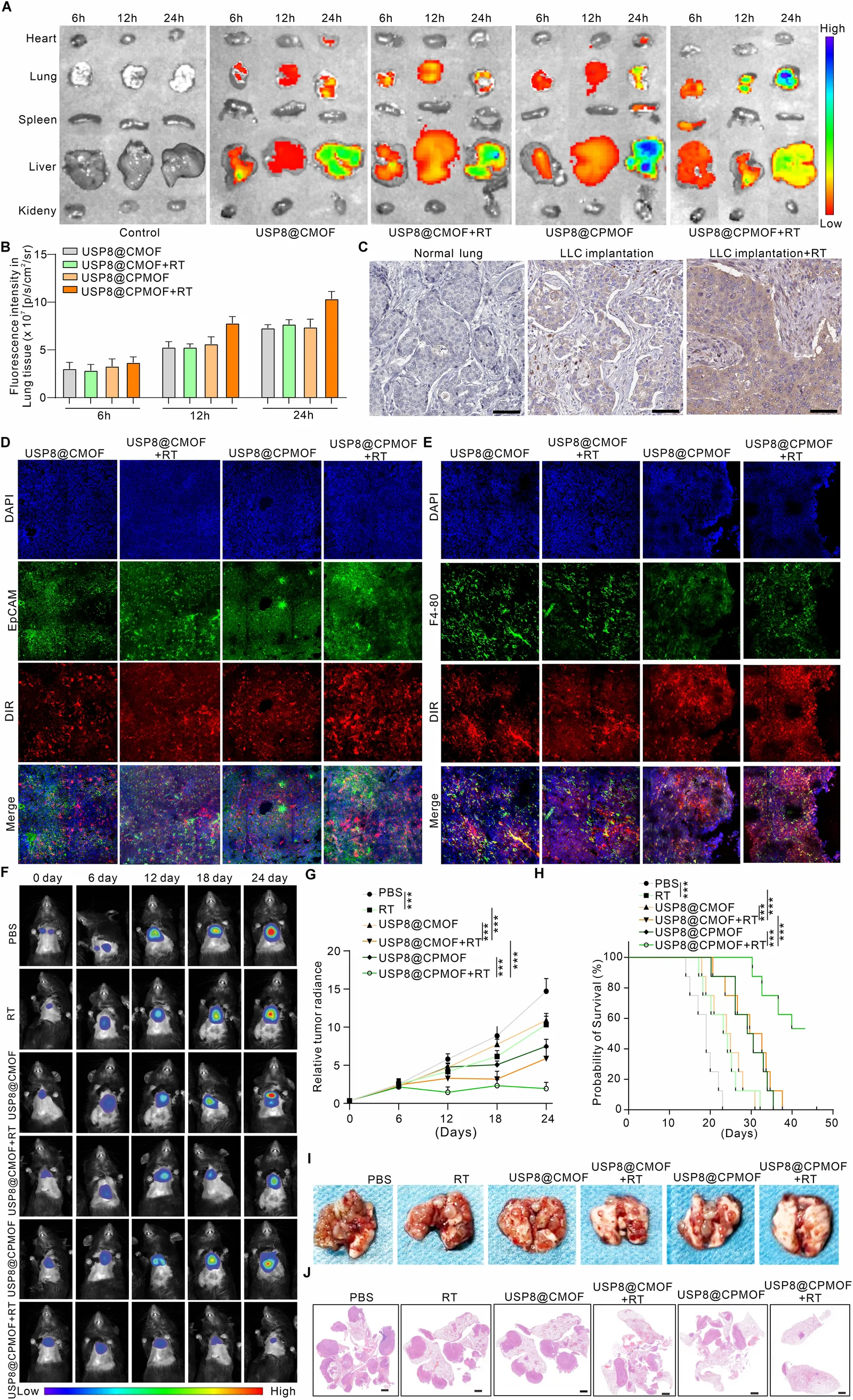
Tumor targeting, infiltration and therapeutic ability by indicated formulations. (A) *Ex vivo* fluorescence imaging was used to identify the distribution in the heart, liver, spleen, lungs (LLC tumor implantation) and kidneys at various time points after intravenous injection in indicated formulations. (B) Quantitative comparison of radiant efficiency to evaluate indicated formulations in the lung tissue (LLC tumor implantation) (n = 3 mice per group). (C) Immunohistochemistry was used to evaluate the upregulation of PD-L1 expression in lung LLC tumors mediated by radiotherapy, the scale bar is 50 μm. (D-E) Immunofluorescence was used *in vivo* to assess the targeting characteristics of indicated formulations for lung cancer tumor cells (D) and macrophages (E) in the lung cancer microenvironment before and after radiotherapy, and the scale bar is 100 μm. (F) *In vivo* bioluminescence imaging was used to evaluate the therapeutic efficacy of indicated treatment groups on the lung metastasis model established with LLC-Luciferase. Radiotherapy was performed one day after intravenous injection in indicated formulations (at 7 days, 13 days, and 19 days). (G) Statistical analysis of relative tumor radiance in different treatment groups at different time points (n = 5 mice per group). (H) Kaplan-Meier survival plot showing survival of mice after the indicated treatments (n = 10 mice per group). (I-J) white images (I) and H&E staining images (J) of lung tumors in different specified treatment groups, and the scale bar is 1 mm. Statistical analysis was performed using one-way ANOVA with Tukey's multiple comparison test. Data are represented as mean ± SEM (NS, not significant, *P < 0.05, **P < 0.01, ***P < 0.001).

We next performed a survival screening study to compare the therapeutic contributions of each functional moiety. Free DUB-IN-2, empty MOF, and anti-PD-L1 antibody alone provided minimal survival benefit, whereas USP8@CPMOF conferred progressively greater survival extension (Fig. S12, Supporting information). Meanwhile, the therapeutic consequence of this targeted delivery was evaluated in an aggressive LLC-luciferase lung metastasis model. *In vivo* bioluminescence imaging showed that while radiotherapy (RT), USP8@CMOF, USP8@CMOF + RT or USP8@CPMOF alone provided modest suppression of tumor growth, their combination (USP8@CPMOF + RT) resulted in the most potent and sustained inhibition of metastatic progression over time (Fig. 5F and G). This superior therapeutic efficacy directly translated into a significant survival benefit, as evidenced by Kaplan-Meier analysis, where mice treated with USP8@CPMOF + RT exhibited the longest overall survival (Fig. 5H). Final validation was provided by macroscopic and histological examination of the lungs. Representative white light images and H&E-stained sections of lung tissues at the study endpoint clearly showed that the USP8@CPMOF + RT combination treatment led to the smallest tumor nodules and the least metastatic burden (Fig. 5I and J), corroborating the bioluminescence and survival data. In conclusion, these *in vivo* findings collectively demonstrate that USP8@CPMOF efficiently homes to irradiated tumor sites, leverages the radiation-induced PD-L1 upregulation for enhanced targeting, and synergizes with radiotherapy to achieve potent anti-tumor activity and significant survival improvement in a preclinical lung metastasis model.

### 2.6 USP8@CPMOF synergizes with radiotherapy to remodel the immunosuppressive tumor microenvironment

To gain comprehensive insights into the immunomodulatory mechanisms of the combination therapy, we performed transcriptomic analysis on tumor tissues. Volcano plot visualization identified numerous differentially expressed genes (DEGs) in the USP8@CPMOF + RT group compared to the RT-alone group (Fig. 6A). KEGG pathway enrichment analysis of these DEGs revealed a significant association with critical ferroptosis-related signaling pathways, including metabolic pathway and biosynthesis of unsaturated fatty acid pathway (Fig. 6B). This was further corroborated by Gene Set Enrichment Analysis (GSEA), which showed a marked positive enrichment of gene sets related to ferroptosis and metabolic pathway in the USP8@CPMOF + RT group (Fig. 6C). These data collectively indicate that the combination therapy robustly activates ferroptosis signaling at the transcriptional level.

**Fig. 6:**
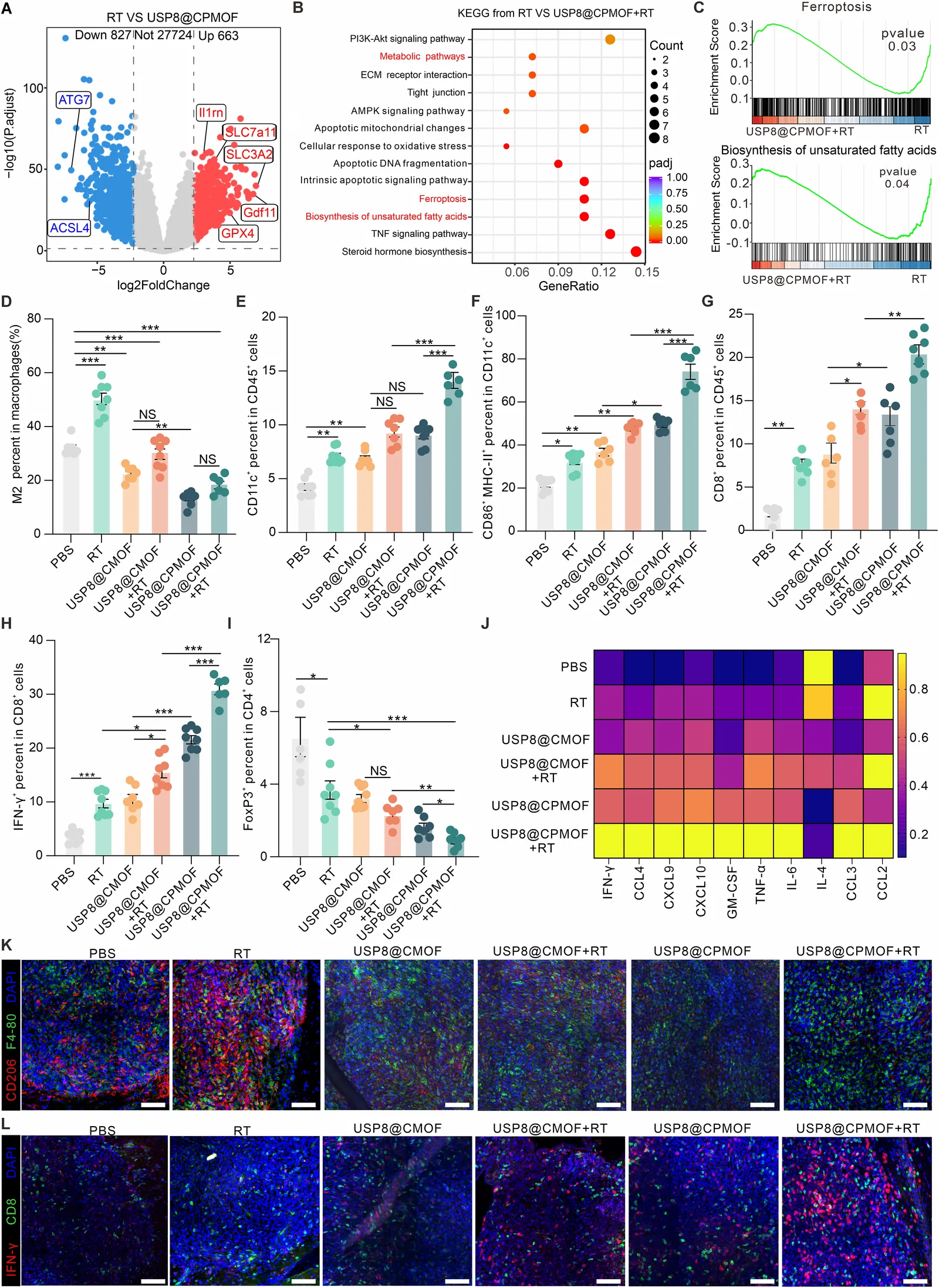
Immunomodulatory effects of indicated formulations with radiotherapy. (A) Volcano plot of differential gene expression in the RT group and USP8@CPMOF + RT group (n = 3 repeats per group). (B) KEGG pathway enrichment analysis in the DEGs between RT group and USP8@CPMOF + RT group. (C) Enrichment analysis of GSEA gene set in USP8@CPMOF + RT group. (D) Percentage of CD206^+^ macrophage among CD45^+^ CD11c^−^ CD11b^+^ F4-80^+^ macrophages. (E) Percentage of CD11c^+^ DCs among CD45^+^ lymphocytes. (F) Percentage of CD86^+^ MHCII^+^ DC cells among CD11c^+^ DCs. (G) Percentages of CD8^+^ T cells among CD45^+^ lymphocytes. (H) Expression percentages of IFN-γ in CD8^+^CD3^+^ T cells across different groups. (I) Percentage of FoxP3^+^ Treg cells among CD4^+^ T cells. (J) ELISAs of cytokine expression levels in different treatment groups. (K) Immunofluorescence staining for F4-80, and CD206 in different treatment groups, and the scale bar is 100 μm. (L) Immunofluorescence staining for CD8, and IFN-γ in different treatment groups, and the scale bar is 100 μm. Statistical analysis was performed using one-way ANOVA with Tukey's multiple comparison test. Data are represented as mean ± SEM (NS, not significant, *P < 0.05, **P < 0.01, ***P < 0.001).

We next characterized changes in the tumor immune landscape using flow cytometry. The combination therapy significantly reduced the proportion of immunosuppressive CD206^+^ M2-like macrophages within the TME (Fig. 6D). Concurrently, it promoted a substantial increase in the infiltration of CD11c^+^ dendritic cells (DCs) (Fig. 6E), and, more importantly, enhanced the maturation status of these DCs, as evidenced by a higher percentage of CD86^+^ MHCII^+^ cells (Fig. 6F). This reshaping of the innate immune compartment was accompanied by a potent activation of adaptive immunity. We observed a significant increase in the infiltration of CD8^+^ T cells (Fig. 6G) and, critically, a higher frequency of IFN-γ-producing cytotoxic T cells within the TME (Fig. 6H). In contrast, the proportion of immunosuppressive FoxP3^+^ regulatory T cells (Tregs) was significantly decreased (Fig. 6I). Furthermore, analysis of cytokine levels in tumor homogenates confirmed a pro-inflammatory shift, with the USP8@CPMOF + RT group showing elevated concentrations of key anti-tumor cytokines such as IFN-γ and TNF-α (Fig. 6J). The flow cytometry findings were visually confirmed by immunofluorescence staining of tumor sections. Staining for F4/80 and CD206 demonstrated a clear reduction in M2-polarized TAMs in the combination treatment group (Fig. 6K). Similarly, staining for CD8 and IFN-γ vividly illustrated enhanced infiltration and functional activation of CD8^+^ T cells within the tumors (Fig. 6L).

In summary, these results demonstrate that USP8@CPMOF synergizes with radiotherapy to orchestrate a comprehensive remodeling of the TME. This multi-faceted immunomodulatory effect includes repolarizing macrophages towards an anti-tumor phenotype, promoting DC maturation and recruitment, enhancing cytotoxic T cell infiltration and function, and suppressing Treg-mediated immunosuppression, thereby converting the TME from immunosuppressive to immunostimulatory.

## 3 Discussion and conclusion

Radiotherapy remains a cornerstone in lung cancer treatment, yet its efficacy is frequently hampered by tumor radioresistance and an immunosuppressive TME. In this study, we identified USP8 as a key regulator of M2 macrophage polarization in the irradiated lung TME and developed a targeted nanoparticle, USP8@CPMOF, to concurrently disrupt this immunosuppressive axis and induce ferroptosis in tumor cells. USP8 is specifically enriched in IL-4/IL-13-induced M2 macrophages and is causally required for maintaining this phenotype, as confirmed by both knockdown and overexpression experiments. Clinical data further support a positive correlation between USP8 and CD206 in lung cancer patients, underscoring the translational relevance of targeting this upstream regulator. The gain-of-function experiments provide complementary evidence that USP8 is not merely associated with but is causally required for M2 polarization, positioning our intervention to temporally precede and enable subsequent T cell activation.

The rational design of USP8@CPMOF was pivotal to translating this mechanistic insight into an effective therapeutic strategy. The core-shell structure, with an iron-based MOF core and a functional cell membrane coating, integrates multiple functionalities: dual-responsive drug release to the acidic and high-GSH TME, and active targeting via an anti-PD-L1 nanobody. This targeting is strictly PD-L1-dependent, as demonstrated by the dramatically reduced uptake in PD-L1 knockout LLC cells both *in vitro* and *in vivo*, and by the abrogation of therapeutic efficacy in PD-L1 KO tumors. This precise targeting is the first step in the causal chain, ensuring that the subsequent release of Fe^2+^ and DUB-IN-2 occurs specifically within the irradiated TME, minimizing off-target effects and maximizing therapeutic efficacy.

The released Fe^2+^ initiates ferroptosis, the first critical event in the cascade. Our results demonstrate that USP8@CPMOF acts as a potent radiosensitizer, amplifying radiation-induced ROS and robustly inducing ferroptosis, as evidenced by lipid peroxide accumulation and labile iron pools. Ferroptosis-specific rescue experiments using Ferrostatin-1 confirm that this pathway is functionally essential for cytotoxicity. Critically, unlike apoptosis, ferroptosis is inherently immunogenic. The robust emission of DAMPs such as CRT and HMGB1 following combination treatment converts tumor cell death into an in situ vaccine, promoting DC maturation and antigen presentation. This establishes ferroptosis as the causal trigger for subsequent immune activation: without ferroptosis-induced DAMP release, DC activation does not occur, and the bridging step from innate to adaptive immunity would be lost.

Concurrent with ferroptosis-induced ICD, the released DUB-IN-2 acts on a distinct cellular compartment—M2-polarized TAMs. USP8@CPMOF directly reprograms immunosuppressive M2 macrophages toward an immunostimulatory M1 phenotype, as demonstrated by CD206 downregulation, CD86 upregulation, and increased secretion of TNF-α, IL-1β, and iNOS. Importantly, free DUB-IN-2 exhibits comparable potency to USP8@CPMOF in macrophage reprogramming, confirming that the effect is specifically driven by USP8 inhibition rather than by the nanoparticle carrier. This parallel event is critically synergistic: while ferroptosis generates the antigens needed for T cell priming, macrophage repolarization dismantles the immunosuppressive barrier that would otherwise constrain T cell activation. The convergence of these two pathways creates a permissive microenvironment where DCs and T cells interact productively, leading to increased CD8^+^ T cell infiltration, IFN-γ production, and reduced Tregs.

Once primed, CD8^+^ T cells infiltrate the tumor and execute their cytotoxic function, completing the cancer-immunity cycle. Importantly, the resulting tumor cell death releases additional tumor antigens, creating a positive feedback loop that sustains and amplifies the anti-tumor response—a feature absent in regimens that induce only apoptosis. This self-amplifying cycle explains the sustained survival benefit observed in our preclinical model. Clinically, USP8@CPMOF shows favorable biodistribution and efficacy without overt toxicity. In conclusion, our work establishes a causal and temporal framework: USP8@CPMOF targeting precedes pH/GSH-responsive drug release, which simultaneously initiates ferroptosis/ICD and macrophage reprogramming; these parallel events converge to prime CD8^+^ T cells, eliminate tumors, and sustain immunity. This study provides a robust foundation for next-generation radio-immunotherapies that tackle multiple pillars of treatment resistance.

## 4 Materials and methods

### 4.1 Materials

Tetramethylbenzidine (TMB) was purchased from Shanghai Macklin Biochemical Co., Ltd. (Shanghai, China). Iron (III) chloride hexahydrate (FeCl_3_⋅6H_2_O) and dithiodiglycolic acid were acquired from Aladdin-Reagent Co. Ltd. (Shanghai, China). β-actin, USP8, HMGB1, CRT, PD-L1, MHC-I and EpCAM antibody were obtained from Cell Signaling Technology (Boston, MA, USA). Secondary antibodies goat anti-mouse IgG H&L-HRP conjugated and goat anti-rabbit IgG H&L-HRP conjugated were bought from Abcam (Cambridge, UK). Hoechst 33,342, ROS assay kit, annexin VFITC apoptosis detection kit and Calcein/PI Cell Viability/Cytotoxicity Assay Kit were obtained from Beyotime Biotechnology Co. Ltd (Shanghai, China). GSH kits were purchased from Nanjing Jiancheng Bioengineering Institute (Nanjing, China). ROS-ID®Hypoxia/Oxidative stress detection kit was obtained from Enzo Life Sciences (Farmingdale, NY, USA). The H2O2 assay kit were purchased from Solarbio (Beijing, China). DUB-IN-2 was purchased from MedChemExpress. The medium of cell culture was purchased from Gibco Life Technologies, Inc. (Grand Island, NY, USA), including Modified Eagle's Medium (DMEM), Roswell Park Memorial Institute (RPMI)-1640 medium. Fetal Bovine Serum (FBS) was obtained from Zhejiang Tianhang Biotechnology Co., Ltd. (Huzhou, China). Plasmocin was bought from InvivoGene (Toulouse, France) and penicillin/streptomycin was obtained from Biosharp (Hefei, China). Sterile 1 × phosphate buffered saline was purchased from Gibco Life Technologies, Inc. (Grand Island, NY, USA). All the cytokines were purchased from Biolegend (San Diego, CA, USA), containing interleukin-4, interleukin-13, and interleukin-10. Acetonitrile, methanol and chloroform for High Performance Liquid Chromatography (HPLC) were all purchased from Thermo Fisher Scientific (Waltham, MA, USA) and their purity was more than 99%. The fluorescence dye DiR were obtained from Yeasen (Shanghai, China). FITC-Liperfluo was obtained from Dojindo (Japan). Phen Green SK diacetate were bought from GLPBIO (USA). Radioimmunoprecipitation assay buffer and the inhibitors of protease and phosphatase were obtained from Beyotime (Shanghai, China). Collagenase IV and hyaluronidase were purchased from Biosharp (Hefei, China). All the antibodies for flow cytometry and immunocyte depletion were bought from Biolegend (San Diego, CA, USA).

### 4.2 Cell cultures

Mouse Lewis cells were obtained from the China Center for Type Culture Collection (Wuhan, China). Cells were grown in Dulbecco's Modified Eagle's Medium containing 10% FBS and 1% penicillin/streptomycin solution. BMDMs were generated as described previously [7,31]. BMDMs were cultured with 20 ng/mL IL-4 and 20 ng/mL IL-13 (210-13-10), to be polarized into IL-4/13 M2 macrophage; 50 ng/mL IL-10 to be polarized into IL-10 M2 macrophage; or 50 ng/mL LPS to be polarized into M1 macrophage. Bone marrow-derived DCs were generated by culturing bone marrow cells from C57BL/6 mice in RPMI-1640 medium supplemented with 10% FBS, 100 ng/ml GM-CSF, and 10 ng/ml IL-4 for 7 days.

### 4.3 Real-time quantitative PCR

Total RNA was harvested from cells using MicroElute Total RNA Kit R6831-01 and reverse-transcribed into cDNA using HiScript III RT SuperMix (+ gDNA wiper). The cDNA was amplified using the AceQ® Universal SYBR qPCR Master Mix on a StepOnePlus Real-Time PCR System. Primer sequences are shown in Supplementary Table S1. All primers were synthesized by Sangon Biotech Co., Ltd (Shanghai).

### 4.4 Western blotting

Cell lysate was prepared for western blotting by lysing cells with RIPA lysis buffer, followed by SDS-PAGE separation and transfer to PVDF membrane. After blocking the membranes for 1 h at room temperature in 5% skim milk powder dissolved in Tris-buffered saline containing 5% Tween-20 (TBST), membranes were incubated overnight at 4 °C with the corresponding antibodies. Then, membranes were washed and incubated with secondary antibodies prior to detection. They were developed using NcmECL Ultra (P10100, NCM Biotech) and imaged.

### 4.5 Transfections

BMDMs were seeded in 6-well-plates (2×105 cells per well). After 24 h, they were transfected with either small interfering RNA (siRNA) against USP8 or negative control (NC) siRNA using Lipofectamine RNAiMAX (Invitrogen, Carlsbad, CA, USA). Cells were harvested after 48 h and processed for western blotting and flow cytometry. USP8 siRNA (sc-76590) and NC siRNA (sc-37007) were purchased from Santa Cruz Biotechnology, Inc.

### 4.6 T-cell proliferation assay

CD8^+^T cells were isolated from the spleens of C57BL/6 mice, following the MojoSort™ Mouse CD8+T Cell Isolation Kit protocol. M2 macrophage induced by IL-4 and IL-13 were treated with DUB-IN-2 for 24 h, and the conditioned medium was collected and filtered through a 0.22 μm filter. Carboxyfluorescein succinimidyl ester-labeled CD8^+^T cells were cultured with IL-2 (20 ng/ml) and stimulated with Dynabeads® Mouse T-Activator CD3/CD28 (11452D, Life Technologies) in the conditioned medium for 4 days. Finally, the proliferation of CD8^+^T cells was detected by flow cytometry.

### 4.7 Mice

Male C57BL/6 J mice (aged 6–8 week, weighted 18–20 g) were purchased from SHULAIBAO Biotech. All mice were kept in micro-isolator cages, and the experimental protocols were approved by the Hubei Provincial Animal Care and Use Committee and were in compliance with the experimental guidelines of the Animal Experimentation Ethics Committee of Wuhan University (No. WP2025038).

### 4.8 Histology, antibody staining, and imaging

To observe different immunocytes in tumor tissue, LLC tumors separated from mice were frozened in optimal cutting temperature (OCT) compound and sectioned followed by paraformaldehyde fixation. Then the slides were blocked by 1% BSA for 20 min at room temperature and incubated with antibodies of F4/80, CD206, CD8 and IFN-γ for half an hour at 4 °C. After washed twice by PBS, 4,6-diamidino-2-phenylindole (DAPI) were added with the slides for nucleus. For lung metastasis assessment, the separated LLC tumors were fixed in 4% paraformaldehyde overnight, dehydrated and embedded in paraffin. The tissues were sectioned and stained by hematoxylin-eosin.

### 4.9 USP8 overexpression in BMDMs

For USP8 overexpression, BMDMs were seeded in 6-well plates at 2 × 10^5^ cells per well and cultured overnight. The cells were then transduced with lentivirus encoding mouse USP8 in the presence of 8 μg/mL polybrene. After 36 h of transduction, the medium was replaced with fresh complete medium containing 2 μg/mL puromycin for stable clone selection. After 72 h of selection, USP8 overexpression was confirmed by Western blotting.

### 4.10 Detection of immunocytes in tumor

LLC tumors from mice were digested into single cell by cutting into small pieces and incubating with Collagenase IV (0.32 mg mL−1) and hyaluronidase (0.5 mg mL−1) for 1 h at 37 °C. The tumor cells were filtered through 70 μm cell strainer after lysis of RBCs. All the samples were blocked Fc receptors followed by incubating with detection antibodies containing CD3 (clone 17A2), CD8 (clone SK1), F4-80 (clone H1.2F3), CD4 (clone GK1.5), FoxP3 (clone 29F.1A12), CD206 (clone 6E6D03), IFN-γ (clone 7F11A10), CD11c (clone N418), CD11b (clone M1/70), CD86 (clone GL-1), MHCII (clone M5/114.15.2), CD45 (clone IM7) and Zombie NIR™, and then measured via flow cytometry.

### 4.11 Preparation of indicated formulations

Synthesis of MOF. Use a peristaltic pump to slowly add FeCl_3._6H_2_O solution (100 mg dissolved in 4 mL of deionized water) to the dithiodiethylene glycol solution (100 mg dissolved in 16 mL of water). Magnetic stirring for about 30 min. When the liquid turns creamy yellow, centrifuge at 8000–10,000 rpm for 10 min to remove the supernatant.

Synthesis of USP8@MOF. Dissolve DUB-IN-2 in DMSO and diluted in water, mix with the above synthetic MOF, and mix with the magnetic force for 24 h. Centrifuge at 10,000 rpm and wash three times for collection. The cell membranes were extracted by liquid nitrogen rapid freezing method and resuspended in PBS solution. The drug-loaded MOF was dissolved in this solution and then sonicated, and press USP8@CPMOF or USP8@CMOF nanoparticles repeatedly. Finally, centrifuge the above nanoparticles (3000 rpm) for 10 min, remove the supernatant, and wash the deionized water 3 times to collect.

### 4.12 Characterization

The morphology and particle size of the nanoparticles were first examined by transmission electron microscopy to visualize their core–shell structure and dispersion. Surface elemental composition were determined by high-resolution X-ray photoelectron spectroscopy. Functional groups and bonding interactions between the core and surface-modifying agents were assessed using Fourier transform infrared spectroscopy.

### 4.13 **Detection the concentration of DUB-IN**-**2 in USP8@CPMOF or USP8@MOF**

DUB-IN-2 concentration within the USP8@CPMOF or USP8@MOF were quantified by high-performance liquid chromatography (HPLC) using an LC-2030C Plus system (Shimadzu, Japan) equipped with a ShimNex C18 column (4.6 × 250 mm, 5 μm, 100 Å). USP8@CPMOF or USP8@MOF were disrupted by adding a 3× volume of acetonitrile and chloroform in a 1:2 (v/v) ratio, vortexed, and centrifuged at 10,000 g for 5 min. The lower organic phase was filtered (0.45 μm PTFE) and injected for analysis. A standard solution of DUB-IN-2 (1 mg) was prepared in the same solvent mixture. Chromatographic separation was performed at 25 °C with a 1 mL min^−1^ flow rate and a mobile phase composed of methanol (A) and acetonitrile (B); detection wavelength was set at 254 nm.

### 4.14 X-ray photoelectron spectroscopy (XPS)

The surface elemental composition and chemical states of MOF, USP8@MOF, and USP8@CPMOF were analyzed by X-ray photoelectron spectroscopy (XPS, Thermo Scientific K-Alpha). Samples were lyophilized and pressed onto indium foil prior to analysis. Survey spectra were acquired in the binding energy range of 0-1200 eV with a pass energy of 200 eV. High-resolution N 1s spectra were collected with a pass energy of 50 eV. Data processing and peak deconvolution were performed using Thermo Avantage software, with binding energy calibration referenced to the C 1s peak at 284.8 eV.

### 4.15 DUB-IN-2/Fe ions release of the USP8@CPMOF

To assess drug release ability, free DUB-IN-2 and USP8@CPMOF were sealed in dialysis bags with a MWCO of 1000 Da, after which the bags were placed in different beakers. An appropriate amount of PBS with pH 5.4 was added to each beaker with or without 10 mM GSH, and all dialysis bags were shaken at 300 rpm and a portion of the supernatant buffer was collected at predetermined time intervals. Drug release was observed by measuring the DUB-IN-2 content with a HPLC method. A similar method was used for the Fe ions release experiments, in which USP8@CPMOF was sealed in dialysis bags with MWCO of 1000 Da, after which the bags were placed in different beakers. An appropriate amount of PBS at pH 7.4 or 5.4 was added to each beaker with or without 10 mM GSH, all dialysis bags were shaken at 300 rpm and a portion of the supernatant buffer was collected at predetermined intervals. Iron ions were measured by using ICP-MS.

### 4.16 Assessment of extracellular and intracellular GSH

The GSH depletion capacity of MOF was first examined outside the cells. 10 mM of GSH solution was mixed with USP8@CPMOF, USP8@CMOF or USP8@MOF (10 mg/mL) and stirred, and the GSH content in the solution was measured at different time points (0, 2, 4, 12 and 24 h), and the GSH solution without MOF was used as the control group. Immediately afterward, the GSH depletion capacity of MOF was assessed intracellularly. LLC tumor cells were seeded in 10 cm cell culture dishes and cultured for 24 h before use. After various treatments, the cells were harvested, washed twice with PBS, then contact sonication at 150 W, the 30 s and repeated 10 times, then centrifuged at 8000 rpm for 10 min. The supernatant was collected and placed in a low-temperature ice bath box at 4 °C, then the change of intracellular GSH content was calculated according to the absorbance value of the enzyme marker according to the instructions book of the GSH kit.

### 4.17 Catalase-like activity assay

The catalase-like activity of the nanoparticles was evaluated using TMB as a substrate. Briefly, USP8@CPMOF, USP8@CMOF, USP8@MOF, or MOF (50 μg/mL) were incubated with H_2_O_2_ (100 μM) and TMB (200 μM) in PBS buffer (pH 6.5) at 37 °C for 30 min. For full-wavelength scanning, the absorption spectra of the reaction mixtures were recorded from 300 to 800 nm using a UV-Vis spectrophotometer (Shimadzu UV-2600). For kinetic analysis, the absorbance at 652 nm was monitored every 2 min over a 40-min period. PBS buffer without nanoparticles was used as the negative control. All experiments were performed in triplicate.

### 4.18 *In vitro* cellular uptake assay

To evaluate the cellular uptake of indicated formulations, different cell lines were planted into 6-well plates and incubated with DiR pre-dyed NPs for 3 or 6 h. The cells were collected, washed by PBS and analyzed through flow cytometry or comfocal imaging.

### 4.19 Immunomodulatory activity of individual carrier components on macrophages

BMDM-derived M2 macrophages (induced by IL-4 and IL-13) were seeded in 6-well plates at 2 × 10^5^ cells per well. The cells were incubated with empty MOF or cell membrane coating alone (at concentrations matching those in USP8@CPMOF) for 48 h. USP8@CPMOF was used as a positive control. After treatment, cells were harvested and stained with anti-CD206 (clone 6E6D03) antibodies, followed by flow cytometry analysis to evaluate changes in M2 marker expression. PBS-treated M2 macrophages served as the untreated control.

### 4.20 Cell viability assay

To evaluate the non-specific cytotoxicity of individual components and radiosensitizing effect of USP8@CPMOF in LLC cells, cell viability was assessed using an CCK8 assay. The cells were cultured in RPMI-1640 or DMEM supplemented with 10% FBS and 1% penicillin/streptomycin at 37 °C in 5% CO_2_. After the cells reached the logarithmic growth phase, they were seeded into 96-well plates (5 × 10^3^ cells/well) and incubated for 24 h until they reached a confluence of ∼60%. USP8@CPMOF were added at various concentrations, followed by radiotherapy after 12 h. After treatment, CCK8 kit reagent was added, and absorbance (650 nm) was measured using a microplate reader.

### 4.21 JC-1 assay, apoptosis detection and ferroptosis detection

JC-1 detection. Cells were cultured in 24-well plates (30,000 cells per well) and then incubated with PBS (control), 50 μg mL−1 USP8@MOF, USP8@CMOF containing 50 μg mL−1 USP8@MOF and USP8@CPMOF containing 50 μg mL−1 USP8@MOF, then with or without 4 Gy X-ray. After treatment, the cells were incubated for 24 h, followed by two washes with PBS to remove residual drugs. JC-1 staining solution (5 μM) was added, and the cells were incubated at 37 °C for 30–60 min to allow the dye to enter the cells and bind to mitochondrial membranes. After incubation, the cells were washed with PBS to remove excess dye and collected for flow cytometry analysis.

Apoptosis detection. Apoptosis was assessed using the Annexin V/PI staining assay. After treatment, the cells were washed twice with PBS, leaving a small volume of buffer at the bottom of the tube. Five microliters of Annexin V-FITC was added to each tube, followed by incubation for 10 min and then the addition of 5 μL of PI solution. After 15 min, 300 μL of PBS was added, and the cells were gently resuspended. The cells were incubated in the dark at room temperature for 10 min before flow cytometry analysis.

Ferroptosis detection. To evaluate ferroptosis, FITC-Liperfluo (5 μM), and Phen Green SK diacetate (10 μM) in 1 mL PBS for 30 min at 37 °C in a cell culture incubator. Subsequently, these cells were washed with PBS twice and resuspended with 200 μL PBS and analyzed via flow cytometry.

### 4.22 Reactive oxygen species (ROS) detection

LLC cells were seeded in confocal dishes at 2 × 10^4^ cells per well and cultured until they reached a confluence of ∼60%. The cells were divided into the same experimental groups as described above. After 24 h of treatment, 10 μM DCFH-DA ROS probe was added to each well, followed by incubation for 30 min. Excess probe was removed by two PBS washes, and 0.5 μg/ml Hoechst 33342 was added to stain nuclei. Fluorescence images were captured using a confocal fluorescence microscope, and ROS generation was assessed by comparing the green fluorescence intensity among the different treatment groups.

### 4.23 C11-BODIPY 581/591 staining for lipid peroxidation

To visualize lipid peroxidation at the cellular level, LLC cells were seeded in confocal dishes at 2 × 10^4^ cells per well and treated as indicated. After treatment, cells were washed twice with PBS and incubated with C11-BODIPY 581/591 (5 μM, Thermo Fisher, D3861) in serum-free medium at 37 °C for 30 min in the dark. After incubation, cells were washed twice with PBS, stained with DAPI (1 μg/mL) for 5 min, and imaged using a confocal laser scanning microscope (Zeiss LSM 880). The fluorescence shift from red (∼590 nm) to green (∼510 nm) was quantified using ImageJ software. For rescue experiments, cells were pre-incubated with Ferrostatin-1 (1 μM, Selleck, S7243) for 2 h before treatment.

### 4.24 FerroOrange staining for labile Fe^2+^ detection

Intracellular labile Fe^2+^ was detected using FerroOrange (Dojindo, F374). LLC cells were seeded and treated as described above. After treatment, cells were washed twice with PBS and incubated with FerroOrange (5 μM) in serum-free medium at 37 °C for 30 min in the dark. Cells were then washed twice with PBS, stained with DAPI, and imaged using a confocal laser scanning microscope. Fluorescence intensity was quantified using ImageJ software.

### 4.25 Ferrostatin-1 rescue assay

To confirm ferroptosis as the major cell death pathway, LLC cells were pre-incubated with Ferrostatin-1 (1 μM, Selleck, S7243) for 2 h before treatment with USP8@CPMOF and/or radiotherapy. For cell viability assessment, cells were seeded in 96-well plates at 5 × 10^3^ cells per well and treated as indicated. After 48 h, cell viability was measured using the CCK-8 assay. For lipid ROS detection, cells were stained with Liperfluo probe as described above, and fluorescence was analyzed by flow cytometry.

### 4.26 Immunogenic cell death (ICD) detection

Cells were seeded at 5 × 10^4^ cells per dish in confocal imaging dishes, treated accordingly, and washed twice with PBS. The cells were fixed in 4% paraformaldehyde for 15 min, followed by three washes with PBS. For HMGB1 detection, the cells were permeabilized with 0.1%–0.3% Triton X-100 for 10 min and then washed three times with PBS. CRT detection was performed without permeabilization. Blocking was performed with 5% BSA or normal goat serum at room temperature for 1 h, followed by an overnight incubation at 4 °C with primary antibodies. After being washed with PBS, the cells were incubated with fluorescent secondary antibodies for 1 h at room temperature in the dark, followed by three washes with PBS. CFSE was used to stain the cell, and DAPI (1 μg/ml) was used to stain nuclei at 37 °C for 5 min, followed by washing with PBS. Imaging was performed using a confocal laser scanning microscope.

### 4.27 Generation of PD-L1 knockout LLC cells using CRISPR-Cas9

PD-L1 knockout LLC cells were generated using the CRISPR-Cas9 system. Briefly, a guide RNA (sgRNA) targeting mouse PD-L1 (5′-TCTTTATATTCATGACCTAC-3′) was cloned into a lentiviral CRISPR/Cas9 expression vector containing a puromycin resistance cassette. Lentiviral particles were produced by co-transfection of HEK293T cells with the CRISPR vector and packaging plasmids (psPAX2 and pMD2.G). LLC cells were transduced with lentiviral particles at a multiplicity of infection (MOI) of approximately 2. Twenty-four hours post-transduction, cells were selected with puromycin (2.5 μg/mL) for 7 days.

### 4.28 *In vivo* antitumor treatment

To establish a lung cancer model based on LLC expressing luciferase, Lewis-luciferase cells (1 × 10^6^ cells suspended in 100 μL of PBS) were administered to mice via intravenous injection. Six days after the inoculation with Lewis-luciferase cells, each mouse underwent bioluminescence imaging to ensure the successful and uniform establishment of the lung metastatic model. Subsequently, the mice were randomly divided into six groups: PBS, RT, USP8@CMOF, USP8@CMOF + RT, USP8@CPMOF, and USP8@CPMOF + RT, and were given their respective treatments (intravenous injection of nanoparticle in 6 day, 12 day and 18 day, RT with total 10 Gy was applied in 7 day, 13 day and 19 day). To assess the development of the lung metastatic model, mice in each group were imaged on the day when all treatments were completed under 1% pentobarbital sodium anesthesia using the Bruker In Vivo MS FX PRO Imager.

### 4.29 In vivo toxicity assessment

To evaluate the biosafety of USP8@CPMOF combined with radiotherapy, C57BL/6 mice bearing LLC tumors were divided into five groups (n = 6 per group): PBS, RT alone, USP8@CMOF, USP8@CMOF + RT, and USP8@CPMOF + RT. Nanoparticles (50 mg/kg) were administered on days 6, 12, and 18; radiotherapy (4 Gy × 3 fractions) was delivered on days 7, 13, and 19. Body weights were recorded every 3 days. At day 21, serum was collected for biochemical analysis of ALT, AST, BUN, and CREA. Major organs (heart, liver, spleen, kidney) were harvested for H&E staining.

### 4.30 Bioluminescence imaging

After anesthetizing C57BL/6J female mice (6∼8 weeks old) with 1% pentobarbital sodium, they were intraperitoneally injected with firefly luciferin (150 mg kg−1; Sigma-Aldrich; CAS: 103404-75-7). After 15 min, mice were imaged using the Bruker In Vivo MS FX PRO Imager with 3 min exposure times for acquiring luminescent images.

### 4.31 Transcriptomic profiling and functional analysis

Total RNA extracted from lung tissues was subjected to library preparation and sequenced on the Illumina platform. Differential gene expression analysis of the bulk RNA-seq data was conducted using DESeq2, which employs a negative binomial model to address read count over-dispersion. Significantly dysregulated genes were defined as those exhibiting |log2FoldChange| > 1 with an adjusted p-value < 0.05 (Benjamini–Hochberg correction). To elucidate the biological implications of the differentially expressed genes, KEGG pathway enrichment analysis was performed using the clusterProfiler package. A hypergeometric test was applied to evaluate the statistical representation of the gene set in each KEGG pathway, with a significance threshold set at p*-*value < 0.05. The results were visualized using dot plots to display enriched pathways based on gene ratio and p*-*value. Special emphasis was placed on pathways related to cytokine-related signaling pathways. Gene Set Enrichment Analysis (GSEA) was performed using the clusterProfiler R package to identify biological pathways exhibiting coordinated expression changes. The analysis was conducted on a pre-ranked gene list sorted in descending order by log2foldchange values. This approach enables the detection of pathways where genes show concordant expression shifts without applying arbitrary significance thresholds. Statistically enriched pathways were identified using an adjusted p-value cutoff of 0.05. The results were visualized using the GseaNb function from the GseaVis package, which effectively displays the enrichment profiles and core enrichment genes for significant pathways.

### 4.32 Statistical analysis

Data were analyzed using GraphPad Prism 9.0 software. Survival rates between groups were compared using the log-rank (Mantel-Cox) test. For comparisons involving three or more groups, one-way analysis of variance (ANOVA) was performed. Differences between two groups were assessed using either a two-tailed unpaired *t*-test or the Mann-Whitney *U* test, depending on data distribution. Statistical significance was set at P < 0.05. Data are presented as means ± standard error of the mean (SEM). Statistical significance is indicated as follows: *P < 0.05; **P < 0.01; ***P < 0.001; ****P < 0.0001; ns, not significant.

## CRediT authorship contribution statement

**Junjian Deng:** Writing – original draft, Methodology, Investigation, Formal analysis, Data curation. **Peiyi Luo:** Investigation, Formal analysis. **Kejun Li:** Methodology, Investigation, Data curation. **Miaomiao Zhang:** Formal analysis, Data curation. **Ruxin Zhang:** Formal analysis, Data curation. **Zengxing Zhang:** Software, Formal analysis. **Songlin Wang:** Writing – review & editing, Supervision, Conceptualization. **Yongfa Zheng:** Writing – review & editing, Supervision, Conceptualization.

## Consent for publication

Not applicable.

## Funding

This work was supported by 10.13039/501100001809National Natural Science Foundation of China (81703035) and Hubei Provincial Natural Science Foundation-Hengrui Pharmaceutical Innovation and Development Joint Fund (2026AFC1206).

## Declaration of competing interest

The authors declare no competing interests.

## Data Availability

Data will be made available on request.
