# Supplementary material for: A USP8-targeting nanoamplifier synergizes with radiotherapy to remodel the immunosuppressive tumor microenvironment in lung cancer
Source: Mater Today Bio. 2026 Sep 4;41:103631. doi: 10.1016/j.mtbio.2026.103631 (PMC13631627; doi:10.1016/j.mtbio.2026.103631)
Supplement: Supplementary file 1 [file mmc1.docx]

**A USP8-Targeting Nanoamplifier Synergizes with Radiotherapy to Remodel the Immunosuppressive Tumor Microenvironment in Lung Cancer**

Junjian Deng^1,#^, Peiyi Luo^1, #^, Kejun Li^1,#^, Miaomiao Zhang，Ruxin Zhang^1^，Zengxing Zhang^2^ , Songlin Wang^3, *^, Yongfa Zheng^1, *^

^1^Department of Oncology, Renmin Hospital of Wuhan University, Wuhan 430060, Hubei, China.

^2^Department of Oncology, Wuxue First People’s Hospital, Wuxue 435401, Hubei, China.

^3^Department of Oncology, Zhongxiang City People’s Hospital, Zhongxiang 431900, Hubei, China.

#These authors contributed equally: Junjian Deng, Kejun Li.

*Corresponding author e-mail: 1436682370@qq.com (Songlin Wang), rm001258@whu.edu.cn (Yongfa Zheng)

**Figure S1. USP8 overexpression promotes M2 polarization.** (**A**) Flow cytometry analysis of CD206 expression in BMDM-derived macrophages following USP8 overexpression. (**B**) Western blot analysis of USP8 and CD206 protein levels. Statistical analysis was performed using unpaired T test. Data are represented as mean ± SD (*n* = 3; NS, not significant, **P* < 0.05, ***P* < 0.01, ****P* < 0.001).

**Figure S2. XPS characterization confirming successful DUB-IN-2 loading and membrane functionalization.** (**A**) Full-scan XPS spectra of MOF, USP8@MOF, and USP8@CPMOF. The N 1s signal (~400 eV) emerges after DUB-IN-2 loading and increases after membrane coating. (**B**) High-resolution N 1s deconvolution spectra. MOF shows no detectable N signal. USP8@MOF exhibits three peaks at 398.5 eV (pyridinic N from DUB-IN-2), 400.0 eV (N-C), and 401.5 eV (protonated N). USP8@CPMOF shows an additional peak at 399.0 eV corresponding to amide N from membrane proteins, confirming successful membrane coating.

**Figure S3**. Western blot analysis using an anti-GFP antibody confirmed the presence of the nanobody-GFP fusion protein (~42kDa) in the cell membrane from anti-PD-L1 nanobody overexpressing cells (Membrane nanobody-OE) and in USP8@CPMOF. Na⁺/K⁺-ATPase served as the membrane loading control, confirming equal membrane protein loading across all membrane-containing lanes.

**Figure S4. Catalase‑like activity of MOF‑based formulations.** (**A**) Full‑wavelength UV‑Vis absorption spectra (300‑800 nm) of the TMB+H₂O₂ reaction system catalysed by different formulations. (**B**) Kinetic curves showing time‑dependent absorbance changes at 652 nm catalysed by different formulations.

**Figure S5. Validation of PD-L1 targeting ability of USP8@CPMOF.** (**A**) Western blot analysis of PD-L1 expression in LLC cells with indicated treatment. β-actin was used as a loading control. (**B**) Representative confocal imaging of USP8@CPMOF to target LLC cells with indicated treatment. The scale bar is 10 μm.

**Figure S6. Individual nanoparticle components have no non-specific cytotoxicity or immunomodulatory activity.** (A) Cell viability of LLC cells treated with individual components at concentrations equivalent to those in USP8@CPMOF. (B) Flow cytometry analysis of M2 macrophages percent after treatment with carrier components. Data are represented as mean ± SEM. Statistical analysis was performed using one-way ANOVA with Tukey's multiple comparison test. NS, not significant, **P* < 0.05, ***P* < 0.01, ****P* < 0.001.

**Figure S7. Ferroptosis validation via cellular imaging and flow cytometry**. (**A**) C11-BODIPY 581/591 staining for lipid peroxidation in indicated treatment group.The USP8@CPMOF+RT group shows a uniform shift from red (reduced) to green (oxidized) fluorescence across all cells, indicating lipid peroxidation. The scale bar is 20 μm. (**B**) FerroOrange staining for labile Fe^2+^  in indicated treatment group. The scale bar is 20 μm. (**C**) Lipid ROS levels in LLC cells infused with indicated treatment measured by Liperfluo probe via flow cytometry (). (D) CCK-8 Cell Viability detection in LLC cells after infused with indicated treatment group. Data are represented as mean ± SEM (n = 3 repeat). Statistical analysis was performed using one-way ANOVA with Tukey's multiple comparison test . NS, not significant, **P* < 0.05, ***P* < 0.01, ****P* < 0.001.

**Figure S8.** USP8 inhibition functionally reprograms M2 macrophages toward an M1-like phenotype. **(A-B)** ELISA quantification of TNF-α (**A**) and IL-1β (**B**) secretion in the supernatants of BMDM-derived M2 macrophages treated with the indicated formulations for 24 hours (n = 5 independent experiments). (**C**) Representative Western blot showing iNOS and β‑actin protein levels in indicated treatment group (n = 3 independent experiments). Data are represented as mean ± SEM. Statistical analysis was performed using one-way ANOVA with Tukey's multiple comparison test . NS, not significant, **P* < 0.05, ***P* < 0.01, ****P* < 0.001.

**Figure S9. PD-L1-dependent tumor targeting of USP8@CPMOF *in vivo***. Representative *ex vivo* fluorescence images of excised lungs from individual mice (n = 3 per group) bearing LLC tumors or PD-L1 knockout LLC tumors after intravenous injection of DiR-labelled USP8@CPMOF. Radiotherapy (RT) was applied to the indicated groups.

**Figure S10**. Body weight changes over 21 days in mice from the five treatment groups (n = 6 per group). No significant differences were observed between any groups at any time point.

**Figure S11.** ***In vivo* toxicity assessment of USP8@CPMOF combined with radiotherapy**. (**A-D**) Serum biochemistry analysis at day 21: (**A**) ALT (alanine aminotransferase), (**B**) AST (aspartate aminotransferase), (**C**) BUN (blood urea nitrogen), and (**D**) CREA (creatinine). (**E**) Representative H&E stained images of major organs (heart, liver, spleen, and kidney) from mice in each treatment group. The scale bar is 100 μm. Data are represented as mean ± SEM (n = 6 mice per group). Statistical analysis was performed using one-way ANOVA with Tukey's multiple comparison test . NS, not significant, **P* < 0.05, ***P* < 0.01, ****P* < 0.001.

**Figure S12**. Kaplan-Meier survival curves comparing seven different treatment groups.

**Supplementary Table 1.** The prime sequence of deubiquitinating enzyme family used in the study.

| Gene | Forward primer | Reverse primer |
| --- | --- | --- |
| UCHL1 | TCTGTCTGAAACGGAGAAGCTGTC | CATGGTTCACTGGAAAGGGCATTC |
| UCHL3 | AGACCAGTATGCGCAGTGTT | TCCAATCGTTCCACAGGCAT |
| UCHL5 | ACAACTTGCAGAGGAACCCAT | CTGGTGCTCAGCTAAGGTCT |
| BAP1 | GAGAGGACGAGGAGTGGACAGATA | CTGCCATCAGGTTGAAGCGAATG |
| JOSD1 | AGACAAGGCCAAATCTGAATCCTT | ATGCTCTTCTTGTGGGGTGTT |
| JOSD2 | GAAGCGAGACCAAGATCACCC | GGGCTAGCCTCTTGCAGATT |
| ATAXIN3 | GTGCTCAGCATTGCCTGAATAACC | TTCTCAGCCTCTCCTCTTCATCCA |
| OTUD1 | CGATTCCACATCATCCCCGA | CCCTCAATGAGTGGGCTGAA |
| OTUD3 | CTTCGTGGAAGATGACATTCCC | AAAGGGGCATTAAGCTGATGG |
| OTUD4 | AGGCAGACATAGACTTGGCTTCAG | TGGTCCTCTTAGGCTCAGGTTCA |
| OTUD5 | AGGACGGTGCCTGTCTATTTC | CATCAGATAGTCCATGCAATGCT |
| OTUD6A | GCCAACTCACCCACCATTGTCA | CCGAATTGTAGTGCTCTCCGAAGT |
| OTUD6B | GCAGCTCACGGAAGATGTTG | AATCCGAGGTGGTTGATTCTCA |
| OTUD7A | ACAGCAGAACAAGGAGGAGGAATG | AGTATGTGGGCGAGGACAAAGAC |
| OTUD7B | CGGAAGATGAGTGGCAGAAGGAA | TGAGCAAGGACGAAGACATGGAAT |
| OTUDB1 | GCTGTGCAGAATCCTCTGGT | AAGCCAAACGCTCGGTAGAA |
| OTUDB2 | AACTCAGCAAAAGATTCACCTCG | TCATTTGGGGTCTGTAGCACA |
| YOD1 | GCGATACTGGGAAAGACAAACG | AGCGGATCGTAGTGAATGCC |
| OTULIN | ACGATGACAAGGAGAAGGGAAAGG | CGGACAGCATAGGCAAGAAGGAA |
| TRABID | TGTGTGGGTGTTGTGGAAGG | GCGTATGGCCAGATGTACCA |
| VCPIP1 | AACCAGAGCACGTCAACACA | CATGCACTAGGCAGTGTCCA |
| PSMD7 | GGACGCCAACGTCAAAAACT | AGTCCCCACTGTAGTGTCCT |
| PSMD14 | AAACATGGTCGTGCTGGAGT | CCAGTTCCTGACTGTGGCAT |
| AMSH | CCCTGAAACCTGGAGCGTTA | AGGTTTCAATGCCTTTGGCG |
| PRPF8 | TGGCCGGAGTGTTTCCTTAC | CATATCGCTTGGCCTGCAAC |
| BRCC3 | GCAGGCGGTTCATCTTGAGT | GACTGTGCGCATTTCCGTTC |
| EIF3H | CACACGGAGGATGATGCTGACTT | TGAGGACGACAGACTCTTCAATGG |
| EIF3F | CACCAACTGCTTTTCGGTGC | TGTCATGGCCTGTGGCATAC |
| MYSM1 | TTCTAATAGCAGCGTCCCCA | ACTCCTCAGCGATGAAGCAA |
| COPS6 | GCCTATGCCCTATGTCACTGTCTT | CCAATGCCTTGTCGGTCGTAGA |
| MPND | AAGAAGATGTGCTGGCTGGG | TTGTCCATACGCTTCCCTGG |
| USP1 | TAATGGGCTTTCAAGAGGCAGT | TCTTCTCGCAGCTTACTGGG |
| USP2 | GTGCTCCACCTGAAGCGATTCT | GGACTTCGGCAGTAGGCTGTATAG |
| USP3 | GTTTGCTCCTTAAGAGATTGCC | TCTCAGTGGGAATTGAACGTAT |
| USP4 | CCAAAAGGTGAAAGGCCAGC | TGCTCAGGTGCTTCTCACAG |
| USP5 | GAAGACGGACAAGACGATGACTGA | ACCTGGACCACGGAGTTGAGATA |
| USP7 | TCGTCGCACATTGAGACGG | CTTGTCGGCATGGTTGGGAAT |
| USP8 | GCCTGCTGTAGCTTCAGTTC | AAGATCTTCTGCGCGCTGTG |
| USP9X | GACCGGGACACCCAAAAGAT | CATGGGGACTTCGCTGACTT |
| USP9Y | AGTTTGCTGTGAAGCCCTGGAA | AGAACTGCTCCTGTGCCAACTG |
| USP10 | CTGAAGCCGTTGAAAAAGATGAG | TCAGCCTCTGCGTTAGAGTTG |
| USP11 | GCAGAACCATAAACGACGAAAT | CACAGATCTGAGATATTGCCCT |
| USP12 | CAGTCTCCAAATTCGCCTCCA | GTGCTCGTTGACCGGAAACT |
| USP13 | CAGTCTGCCCTTGGCATTTAC | CCTCTTCTCTTAGGGAGCACAG |
| USP14 | ACCTCCAATGGTGTTCAAAGC | CATCCTTCAGGGTTCCTCCTT |
| USP15 | TCAGCCATTCAGTGTTTGAGC | TTTCACCTCTCATTCCTAAGGGA |
| USP16 | TGGCTCCTTTTTGTACCCTTAA | GTACCACTGTGTTCAACAACTC |
| USP18 | CAGGAGTCCCTGATTTGCGTG | CAGAGGCTTTGCGTCCTTATC |
| USP19 | TGGAGATGCTAGGAGAGTGTC | CGCAGCTTAACAATCACCTCAT |
| USP20 | TGGACTGCATAGGGGAGGTG | GGCAAGCCCATAGGTTAGGTC |
| USP21 | AACTCCATGTTACGACCTTTGC | AAGGGGACCTCTAGGACGAGA |
| USP22 | TCTTTCTGTCGGATAGGCACC | GCCCTGAGTAAAACTCCTGGA |
| USP24 | GCTGGAAAGCCGCGTTTTG | CAAGTCTGGCTAAGTAGGTGGA |
| USP25 | TCTCGAAACCCCTATGACAGAA | TGAATAACTGCACTAAACCAGCA |
| USP27X | GACATTGAGCAAATTGCCAAAGA | AAGTCCCGGCACTGAACAC |
| USP28 | GGGTCCGAGAAGGAAAGCC | CACGGAACGATCCGAAGGAAG |
| USP29 | GCTGGGTGATAATGTTACAGGC | TGAGTAAGGATGTGTCGTCTCT |
| USP30 | CCTCACCCCACGACCAATC | GAGAAAGGCTGTCAAAGGTGT |
| USP31 | TTCTTTGCCAATTCCTCTACCC | CACAGCCACACCGATCCTC |
| USP32 | GTCCCAGATACACTCAGGAAGT | AGCGAGAGAAGGTAAAAGCATC |
| USP33 | GGACCACAGCACCATACACTC | GCTGCAAGCATAACACCATACT |
| USP34 | CTGGTTGCCTATGAAGGCTTG | AGCTTGATGCAGTTTTTCGACA |
| USP36 | CCAACAGCGGCAATGCTATC | CATCGCATCAATGGTGTACCG |
| USP37 | AGTCAGCCTGCTCGTTCACTA | AAGTCAACATTAGGCGGCTTT |
| USP38 | GCCCCTCAAGCGGATGATT | GGGTCGTCAGGTCAAACATGG |
| USP39 | GTCACTGCCCGTACTTGGATA | GTATGCGTTGATGTGCGAGAG |
| USP40 | TGACCGACTGGTTAAAGCAGC | GCTAGTATCCTTGTAGCGTTCAC |
| USP42 | AGGCGGTCTCACCTGAAGA | CACTGGCCCTAATGGAAGTGT |
| USP45 | ATGCGGGTAAAAGATCCATCAAA | ACGTTAGACCTACAGCAATGTCA |
| USP46 | ATGACTGTCCGAAACATCGCC | TTGACCAATCCGAAGTAGTGTTC |
| USP47 | GATGTGATTCCCTTGGATTGCT | AACCCCATTGGTGTATCTTCTTC |
| USP48 | CAGAGGAAACCCGAATTGCTT | GTGGCTCCCAGGTTAGTCAAG |
| USP49 | AGTTCCGGGAATGTTTCCTGA | CTCCTTACTGACAACTCTGCG |
| USP26 | ACCAATGAGGACCACAACCA | CACTCTGGTTCACACCCCTT |
| USP35 | GGAGTGGGTTCTAAGGCAAGAA | GTAGGACGAAGTCACCACGG |
| USP43 | GCAGGCCCGAGGTTCATC | TGTGAGGTGCCAGCTGTACT |
| USP44 | CCGTGCCTGGTTACTGACAT | CTGCTTCTGGGCTTCTGTGA |
| USP50 | CCTCTGCAGCGTATCTCCAC | TCATCAGGTAGGCAAAGGCG |
| USP51 | TGCGGATGATCTATCAGCGTTTCA | CACAGGACAGACAAGAGTGGAGTC |
| USP52 | GGCTTCCTCTTCCACATGCT | AGAAACTGCCTCCAGCACC |
| USP53 | CTGTTACACCAGCCGACACT | AAGGGGCTGTAAGTGGCATC |
| USP54 | GCAGAATCAGGGGAGGAGTG | TTACACAGAGCCAGAGCTGC |
| CYLD | ACCCTACTGGGAAGAACGGAT | CGGTCTTGGATGTACTGTCCTAT |
| USP17le | GGTCTTTGGAGACATGGTGGT | TCTGGGGCATCAGGAGATGA |
| USPL1 | CAGGGACTGATGTAGGGATATCTT | GTGACTCAGAGCCCAAAGGAT |
| DESI2 | AGATTCCTCGCTGGATCAACCG | GGCTGCATCCTCTGCTTCTTCT |
